# Supplementary material for: A mechanistic and data-driven reconstruction of the time-varying reproduction number: Application to the COVID-19 epidemic
Source: PLoS Comput Biol. 2021 Jul 26;17(7):e1009211. doi: 10.1371/journal.pcbi.1009211 (PMC8341713; doi:10.1371/journal.pcbi.1009211)
Supplement: S1 Text — (PDF) [file pcbi.1009211.s001.pdf]

A mechanistic and data-driven reconstruction of the time-varying reproduction number: Application to the COVID-19 epidemic

Bernard Cazelles[a,b,c], Clara Champagne[d,e], Benjamin Nguyen-Van-Yen[c,f], Catherine Comiskey[g], Elisabeta Vergu[b,☯], Benjamin Roche[h,☯]

a. Sorbonne Université, UMMISCO, Paris, France
b. INRAE, Université Paris-Saclay, MaIAGE, Jouy-en-Josas, France
c. Eco-Evolution Mathématique, IBENS, UMR 8197, CNRS, Ecole Normale Supérieure, Paris, France
d. Swiss Tropical and Public Health Institute, Basel, Switzerland
e. Universty of Basel, Basel, Switzerland
f. Institut Pasteur, Unité de Génétique Fonctionnelle des Maladies Infectieuses, Paris, France
g. School of Nursing and Midwifery, Trinity College Dublin, The University of Dublin, Dublin, Ireland
h. MIVEGEC, IRD, CNRS and Université de Montpellier, Montpellier, France
☯ These authors contributed equally to this work.

Supporting Information

State-space framework

We have used a state-space framework defined by two sets of equations: the first set describes the propagation of the disease in the population and the second corresponds for the observation process. This allows to consideration of unknowns and uncertainty both in the epidemiological process and in the observation of epidemic dynamics:

$$\begin{cases} \dot{x}(t) = g\left(t, x(t), \theta(t), u(t)\right) \\ y(t) \mid x(t) \sim f\left(h(x(t)), \phi(t)\right) \end{cases} \tag{S1}$$

The first equation of (S1) corresponds to the epidemiological model, with *x(t)* representing the state variables (for instance, $S(t)$ the susceptibles, $E(t)$ the infected non-infectious, $I(t)$ the infectious and $R(t)$ the removed for the classical SEIR model) and $\theta(t)$ the epidemiological parameters, among which some are time-dependent. The second equation is associated to the observational process defined by the probabilistic distribution f with parameters depending on a functional of some of the epidemiological model coordinates, $h(x(t))$ and some observational and/or measurement error, $\phi(t)$. $y(t)$ corresponds to observed epidemic dynamics (partial and noisy observations of $x(t)$) and $u(t)$ is the process noise describing intrinsic or environmental stochasticity other than the observational noise is included in f. In our applications, $h(x(t))$ will represent the daily new cases.

Epidemiological Model – hidden state

Our objective was to propose a simple but relevant model that incorporates the available information to examine the dynamics of Covid-19 epidemics before, during and after the lock-down.

Our model is an extended stochastic SEIR model also accounting for asymptomatic transmission and the hospital course of the hospitalized infectious. It is similar to other models that have already been proposed to model and forecast the Covid-19 epidemic [1-5]. It includes the following variables the susceptibles *S*, the infected non-infectious *E*, the symptomatic infectious *I*, the asymptomatic infectious *A*, the removed people *R*, and the hospital-related variables: hospitalized people *H*, people in intensive care unit *ICU*, cured people *G*, and deaths at hospital *D*. We have also introduced Erlang-distributed stage durations (with a shape parameter equal to 2) in *E*, *I*, *A* and *H* compartments, to relax the mathematically convenient but unrealistic assumption of exponential stage durations. As more and more people have been vaccinated and as the duration of the immunity conferred shows a good persistence of the antibodies induced [6], the effect of vaccination is introduced simply by considering the effect of vaccination on the depletion of susceptibles. For sake of simplicity, we present here below the differential equations describing the deterministic version of our model, whereas its stochastic version was used in this study:

$$\frac{dS}{dt} = -\beta(t).\left(I_1 + q_1.I_2 + q_2.(A_1 + A_2)\right) - V$$
$$\frac{dE_1}{dt} = \beta(t).\left(I_1 + q_1.I_2 + q_2.(A_1 + A_2)\right) - \frac{\sigma}{2}.E_1$$
$$\frac{dE_2}{dt} = \frac{\sigma}{2}.(E_1 - E_2)$$
$$\frac{dI_1}{dt} = (1 - \tau_A).\frac{\sigma}{2}.E_2 - \frac{\gamma}{2}.I_1$$
$$\frac{dI_2}{dt} = \left(1 - \tau_H - q_I.\tau_I\right)\frac{\gamma}{2}.I_1 - \frac{\gamma}{2}.I_2$$
$$\frac{dA_1}{dt} = \tau_A.\frac{\sigma}{2}.E_2 - \frac{\gamma}{2}.A_1$$
$$\frac{dA_2}{dt} = \frac{\gamma}{2}.(A_1 - A_2)$$
$$\frac{dR}{dt} = \frac{\gamma}{2}.\left(\left(1 - \tau_H - q_I.\tau_I\right).I_2 + A_2\right) + (1 - q_D.\tau_D).\frac{\kappa}{2}.H_2 + V \tag{S2}$$
$$\frac{dH_1}{dt} = \tau_H.\frac{\gamma}{2}.\left(I_1 + I_2\right) - \frac{\kappa}{2}.H_1$$
$$\frac{dH_2}{dt} = \left(1 - \tau_I - q_D.\tau_D\right).\frac{\kappa}{2}.H_1 + (1 - \tau_D).\delta.ICU - \frac{\kappa}{2}.H_2$$
$$\frac{dICU}{dt} = q_I.\tau_I.\frac{\gamma}{2}.\left(I_1 + I_2\right) + \tau_I.\frac{\kappa}{2}.H_1 - \delta.ICU$$
$$\frac{dG}{dt} = \left(1 - q_D.\tau_D\right).\frac{\kappa}{2}.H_2$$
$$\frac{dD}{dt} = q_D.\tau_D.\frac{\kappa}{2}.\left(H_1 + H_2\right) + \tau_D.\delta.ICU$$

The main originality of this model is the time-varying transmission rate *β(t)* that follows a Brownian diffusion process, equation (1) in the main text. σ is the incubation rate and $1/\sigma$ is the average incubation period. As in previous models, *e.g.* [3,7], we reduce the incubation period duration to account for pre-symptomatic infectious. In this way individual that become infectious before the end of the infectious period but remain either asymptomatic or develop symptoms are put in the infectious compartment. The other parameters are: γ the recovery rate, $1/\kappa$ the average hospitalizion period, $1/\delta$ the average time spent in ICU, τ_A the fraction of asymptomatics, τ_H the fraction of infectious hospitalized, τ_I the fraction of ICU admission, τ_D the death rate, q_1 and q_2 the reduction in transmissibility of I_2 and asymptomatics (A_1+A_2), respectively. As the peaks of hospitalized and admitted to ICU individuals are concomitant, we consider that a small fraction, $q_I.\tau_I$ of infectious with severe symptoms enter directly the ICU. Even if the majority of deaths occur in the ICU, a fraction, $q_D.\tau_D$, can occur in the hospital, outside the intensive care. Then, q_I and q_D are the reduction in admission in ICU and in death rate, respectively. Most of the parameters are estimated in our inference process and the remaining ones set to plausible values, in agreement with the literature (see S1 Table). In this model, variable V represents vaccinated

people and can be interpreted as "effectively protected vaccinated people". V is proportional to the number of individuals vaccinated with one and/or two doses as:

$$V(t) = e_1.V_{dose1}(t-d_e) + e_2.V_{dose2}(t-d_e) \qquad \text{(S3)}$$

where V_{dosei} is the number of individuals vaccinated with one or two doses, d_e the delay after vaccination and e_i is the vaccine efficiency. For this latter parameter, the range documented in the literature is between [17%-73%] for one dose and [52 %-93%] for 2 doses [8-12], by considering all types of available vaccines. Of course, the vaccine efficiency depends on population analyzed, the vaccine used and the delay after vaccination. The delay before protection varies according to the studies between 1 week and 5 weeks [8-12]. For the AstraZeneca/Oxford only the effect of one dose has been studied in UK [8]. Taking these analyses into consideration we used conservative values: $e_1 = 0.45$; $e_2 = 0.85$ and $d_e = 14$ days.

Our inference approach is mainly based on data on new entries in several model compartments. These observed variables used are described in the following equations:

$$\begin{aligned}
C_I(t) &= \int_t^{t+1} (1-\tau_A).\frac{\sigma}{2}.E_2(t).dt \\
C_H(t) &= \int_t^{t+1} \tau_H.\frac{\gamma}{2}.\left(I_1(t)+I_2(t)\right).dt \\
C_{ICU}(t) &= \int_t^{t+1} \left(q_I.\tau_I.\frac{\gamma}{2}.\left(I_1(t)+I_2(t)\right) + \tau_I.\frac{\kappa}{2}.H_1(t) \right).dt \qquad \text{(S4)} \\
C_G(t) &= \int_t^{t+1} \left(1-q_D.\tau_D\right).\frac{\kappa}{2}.H_2(t).dt \\
C_D(t) &= \int_t^{t+1} \left(q_D.\tau_D.\frac{\kappa}{2}.\left(H_1(t)+H_2(t)\right) + \tau_D.\delta.ICU(t) \right).dt
\end{aligned}$$

where C_I is the incidence of symptomatic cases, C_H the daily admission to hospital, C_{ICU} the daily new admission in ICU, C_D the new daily number of of hospital deaths and C_G the daily new discharge at hospital.

Observational Model

Our model trajectories are related to the data using a negative binomial observation model, which is commonly used [13]. The observed incidences, $C_{k,obs}(t)$, are assumed to be drawn from a negative binomial distribution with mean $\rho_k.C_k(t)$ and variance $\rho_k.C_k(t) + \phi_k.(\rho_k.C_k(t))^2$. For each k, $C_k(t)$ represents the number of events occurring during day t simulated by the model, $\rho_k \in [0,1]$ is an observation rate (or reporting rate) and ϕ_k is an over-dispersion parameter.

Current hospital data, $H_{obs}(t)$ (corresponding to H_1+H_2+ICU) and $ICU_{obs}(t)$ (corresponding to ICU), are also available and therefore used to fit the model. We make the assumption that these variables $X_{k,obs}$ follow a discretized normal distribution with fixed standard deviation [14]. In a first approximation, we assume that the mean equals the predicted values by the model X_k (equations S2) times an observation rate ρ_k (see Table S1) [14] and that the fixed standard deviation is proportional to its estimated highest value.

All variables are assumed to be independent conditional on the underlying epidemiological model, and therefore the model likelihood is the product of the likelihoods for all observed variables.

Inference

Stochastic framework

Equations (S1-S4) are considered in a stochastic framework solved with the Euler-Maruyama algorithm [15] implemented in the SSM platform [16].

Estimation using the particle Markov Chain Monte Carlo (PMCMC) method

The likelihood of our stochastic model being intractable, it was estimated with particle filtering method (Sequential Monte Carlo, SMC). With a given set of parameters, the SMC algorithm reconstructs sequentially the trajectory of the state variables and the time-varying parameters, and allows to compute the associated likelihood. Firstly, the distribution of the initial conditions of the system is approximated by a sample of particles. Then, at each iteration, the particles are projected according to the epidemiological and observation models up to the next observation point. To each of the particles a weight is associated, reflecting the quality of its prediction compared to the observation, and the total likelihood is updated. A resampling step using the weights is performed before the next iteration, in order to discard the trajectories associated with low weight particles.

In order to estimate the parameters of the system, the particle filter is embedded in a Markov Chain Monte Carlo framework, leading to the PMCMC algorithm [17]. More precisely, the likelihood estimated by the SMC method is used within a Metropolis Hasting scheme (particle marginal Metropolis Hastings) [17]. The proposal distribution is a Gaussian whose co-variance matrix is adapted following the framework described in Dureau et al [16].

The starting point of the MCMC chain is initialized using optimal values obtained from a simplex algorithm adapted for stochastic system (KSimplex), on a large number of parameter sets. Then, a pre-adaptation of the proposal co-variance matrix is performed with Kalman MCMC (KMCMC). The main idea behind these algorithmic improvements relies on less computationally costly algorithms in order to facilitate the exploration of parameter space. As our model is not Gaussian, we approximate the likelihood using the extended Kalman filter both in the simplex algorithm (KSimplex) [16] and in the MCMC (KMCMC) step [18]. Then, the adaptive PMCMC is run on the output of the KMCMC step with 100,000 iterations and 2,000 particles for the estimates showed in figures included in the manuscript.

PMCMC algorithm as implemented in SSM (Dureau et al, 2013b)

In a model with n observations and J particles.
$q(.|\theta^{(i)})$ is the transition kernel

1. Initialize $\theta^{(0)}$
2. Using SMC algorithm, compute $\hat{p}(y_{1:n}|\theta^{(0)})$ and sample $x_{0:n}^{(0)}$ from $\hat{p}(x_{0:n}|y_{1:n}, \theta^{(0)})$
3. **for** $(i = 0: N - 1)$ **do**
4. Sample θ^* from $q(.|\theta^{(i)})$
5. Using SMC algorithm, compute $L(\theta^*) = \hat{p}(y_{1:n}|\theta^*)$ and sample $x_{0:n}^*$ from $\hat{p}(x_{0:n}|y_{1:n}, \theta^*)$
6. Accept θ^* (and $x_{0:n}^*$) with probability $1 \wedge \frac{L(\theta^*)p(\theta^*)q(\theta^{(i)}|\theta^*)}{L(\theta^{(i)})p(\theta^{(i)})q(\theta^*|\theta^{(i)})}$
7. If accepted, $\theta^{(i+1)} = \theta^*$ and $x_{0:n}^{(i+1)} = x_{0:n}^*$. Otherwise, $\theta^{(i+1)} = \theta^{(i)}$ and $x_{0:n}^{(i+1)} = x_{0:n}^{(i)}$.
8. **end for**

Then, the adaptive PMCMC is run on the output of the KMCMC. For each region or country just one chain of PMCMC has been run with p particles m iterations for the chain. Depending on the convergence velocity, we have used p=2000 or p=3000 particles with m=100,000 or m=200,000 iterations. Each time the burning is equal to 20% of the length of the analyzed chain m. In order to assess convergence of the chains, the visual inspection of the chains (see S2 Fig.) was complemented by Geweke diagnosis [19] (Geweke, 1992), the parameter means computed using the first 20% and the last 50% of the chain are compared through a Z-score.

References

1. Li R, Pei S, Chen B, Song Y, Zhang T, Yang W, Shaman J. Substantial undocumented infection facilitates the rapid dissemination of novel coronavirus (SARS-CoV-2). Science. 2020;368:489-493

2. Prague M, Wittkop L, Clairon Q, Dutartre D, Thiébaut R, Hejblum BP. Population modeling of early COVID-19 epidemic dynamics in French regions and estimation of the lockdown impact on infection rate. MedRxiv. 2020;2020.04.21.20073536.

3. Di Domenico L, Pullano G, Sabbatini CE, Boëlle PY, Colizza V. Impact of lockdown on COVID-19 epidemic in Ile-de-France and possible exit strategies. BMC Medicine. 2020;18:240.

4. Liu Z, Magal P, Webb G. Predicting the number of reported and unreported cases for the COVID-19 epidemics in China, South Korea, Italy, France, Germany and United Kingdom. J Theor Biol. 2020;509:110501.

5. Prem K, Liu Y, Russell TW, Kucharski AJ, Eggo RM, Davies N et al. The effect of control strategies to reduce social mixing on outcomes of the COVID-19 epidemic in Wuhan, China: a modelling study. The Lancet Public Health. 2020;5:e261-e270.

6. Widge AT, Rouphael NG, Jackson LA, Anderson EJ, Roberts PC, Makhene M, Chappell JD, Denison MR, Stevens LJ, Pruijssers AJ, McDermott AB, Flach B, Lin BC, Doria-Rose NA, O'Dell S, Schmidt SD, Neuzil KM, Bennett H, Leav B, Makowski M, Albert J, Cross K, Edara VV, Floyd K, Suthar MS, Buchanan W, Luke CJ, Ledgerwood JE, Mascola JR, Graham BS, Beigel JH; mRNA-1273 Study Group. Durability of Responses after SARS-CoV-2 mRNA-1273 Vaccination. N Engl J Med. 2021;384(1):80-82.

7. Ferguson, N. M., Laydon, D., Nedjati-Gilani, G., Imai, N., Ainslie, K., Baguelin, M., ... & Van-Elsland, S. Impact of non-pharmaceutical interventions (NPIs) to reduce COVID-19 mortality and healthcare demand. Imperial College COVID-19 Response Team. 16 March 2020.

8. Bernal JL, Andrews N, Gower C, Stowe J, Robertson C, Tessier E, Simmons R, Cottrell S, Roberts R, O'Doherty M, Brown K, Cameron C, Stockton D, McMenamin J, Ramsay M. Early effectiveness of COVID-19 vaccination with BNT162b2 mRNA vaccine and ChAdOx1 adenovirus vector vaccine on symptomatic disease, hospitalisations and mortality in older adults in England. MedRxiv. 2021; 2021.03.01.21252652.

9. Dagan N, Barda N, Kepten E, Miron O, Perchik S, Katz MA, Hernán MA, Lipsitch M, Reis B, Balicer RD. BNT162b2 mRNA Covid-19 Vaccine in a Nationwide Mass Vaccination Setting. N Engl J Med. 2021;384:1412-1423.

10. Hall VJ, Foulkes S, Saei A, Andrews N, Oguti B, Charlett A, Wellington E, Stowe J, Gillson N, Atti A, Islam J, Karagiannis I, Munro K, Khawam J, Group The SIREN Study, Chand MA, Brown C, Ramsay ME, Bernal J, Hopkins S. Effectiveness of BNT162b2 mRNA vaccine against infection and COVID-19 vaccine coverage in healthcare workers in England, multicentre prospective cohort study (the SIREN Study). The Lancet Preprints. 2021: Available at SSRN: https://ssrn.com/abstract=3790399 or http://dx.doi.org/10.2139/ssrn.3790399

11. Moustsen-Helms IR, Emborg HD, Nielsen J, Nielsen KF, Krause TG, Molbak K, Møller KL, Berthelsen ASN, Valentiner-Branth P. Vaccine effectiveness after 1st and 2nd dose of the BNT162b2 mRNA Covid-19 Vaccine in long-term care facility residents and healthcare workers–a Danish cohort study. MedRxiv. 2021;2021.03.08.21252200.

12. Thompson MG, Burgess JL, Naleway AL, Tyner HL, Yoon SK, Meece J, Olsho LEW, Caban-Martinez AJ, Fowlkes A, Lutrick K, Kuntz JL, Dunnigan K, Odean MJ, Hegmann KT, Stefanski E, Edwards LJ, Schaefer-Solle N, Grant L, Ellingson K, Groom HC, Zunie T, Thiese MS, Ivacic L, Wesley MG, Lamberte JM, Sun X, Smith ME, Phillips AL, Groover KD, Yoo YM, Gerald J, Brown RT, Herring MK, Joseph G, Beitel S, Morrill TC, Mak J, Rivers P, Harris KM, Hunt DR, Arvay ML, Kutty P, Fry AM, Gaglani M. Interim estimates of vaccine effectiveness of BNT162b2 and mRNA-1273 COVID-19 vaccines in preventing SARS-CoV-2 infection among health hare personnel, first responders, and other essential and frontline workers - eight U.S. locations, December 2020-March 2021. MMWR Morb Mortal Wkly Rep. 2021;70:495-500.

13. Bretó C, He D, Ionides EL, King AA. Time Series Analysis via Mechanistic Models. The Annals of Applied Statistics 2009;3:319-348.

14. Davies NG, Barnard RC, Jarvis CI, Russell TW, Semple MG, Jit M, Edmunds WJ; Centre for Mathematical Modelling of Infectious Diseases COVID-19 Working Group; ISARIC4C investigators. Association of tiered restrictions and a second lockdown with COVID-19 deaths and hospital admissions in England: a modelling study. Lancet Infect Dis. 2021;21:482-492.

15. Kloeden P, Platen E. Numerical Solution to Stochastic Differential Equations. 1999. Springer.

16. Dureau J, Ballesteros S, Bogich T. SSM: Inference for time series analysis with State Space Models. 2013. https://github.com/JDureau/ssm/blob/master/doc/doc.pdf.

17. Andrieu C, Doucet A, Holenstein R. Particle markov chain monte carlo methods. Journal of the Royal Statistical Society: Series B. 2010;72:269-342.

18. Dureau J, Kalogeropoulos K, Baguelin M. Capturing the time-varying drivers of an epidemic using stochastic dynamical systems. Biostatistics. 2013;14: 541-555.

19. Geweke, J. Evaluating the accuracy of sampling-based approaches to calculating posterior moments. In Bernado JM, Berger JO, Dawid AP, Smith AFM, editors. Bayesian Statistics 4. Clarendon Press, Oxford, UK;1992.

S1 Table. Definition of the different parameters and their priors for Ile-de-France region, Ireland and four other French regions: Provence Alpes Côte d'Azur (PACA), Occitanie (OC), Nouvelle-Aquitaine (NA), Auvergne Rhône Alpes (ARA). The priors have been chosen based on current literature [1-5,7].
U stands for uniform distribution and tN for truncated normal distribution (tN[mean,std,limit inf,limit sup]).

**Parameters**	**Definitions**	**Prior or constant value**	**Prior or constant value**	**Prior or constant value**
		**Ile de France***	**Ile de France****	**Ireland**
$I_1(0)$	Initial condition	U[10,1500]	U[10,1500]	U[5,100]
$S(0)$	Initial condition	N=12278000	N=12278000	N=5176000
$E_1(0)$, $E_2(0)$, $I_2(0)$, $A_1(0)$, $A_2(0)$	Initial conditions	Use of steady-state conditions ***	Use of steady-state conditions ***	Use of steady-state conditions ***
$H_1(0)$, $H_2(0)$, $ICU(0)$, $D(0)$, $G(0)$, $R(0)$	Initials conditions	0	0	0
$\beta(0)$	Initial condition of the transmission rate	0.85	0.85	0.70
ν	Volatility of the Brownian process	U[0.02,0.15]	U[0.02,0.15]	U[0.05,0.15]
$1/\sigma$	average duration of the incubation period	tN[4,0.1,3,5] (Di Domenico et al, 2020)	tN[4,0.1,3,5] (Di Domenico et al, 2020)	tN[4,0.1,3,5] (Di Domenico et al, 2020)
$1/\gamma$	average duration of the infectious period	tN[6,0.2,4.5,7.5] (eg Ferguson et al, 2020)	tN[6,0.2,4.5,7.5] (eg Ferguson et al, 2020)	tN[6,0.2,4.5,7.5] (eg Ferguson et al, 2020)
$1/\kappa$	average hospitalization period	U[10,20]	U[10,20]	U[8,20]
$1/\delta$	average time spent in ICU	U[10,20]	U[10,20]	U[8,20]
τ_A	fraction of asymptomatics	U[0.30,0.70]	U[0.30,0.70]	U[0.30,0.70]
τ_H	fraction of hospitalization	U[0.02,0.10]	U[0.02,0.10]	U[0.02,0.10]
τ_I	fraction of ICU admission	U[0.05,0.15]	U[0.05,0.15]	U[0.025,0.15]
τ_D	death rate	U[0.10,0.80]	U[0.10,0.80]	U[0.10,0.60]
q_1	reduction in transmissibility	1.5*q_2 but≤1	1.5*q_2 but≤1	1.5*q_2 but≤1
q_2	reduction in transmissibility	0.55 (Li et al, 2020)	0.55 (Li et al, 2020)	0.55 (Li et al, 2020)
q_I	reduction in ICU admission fraction	0.05	0.05	0.10
q_D	reduction in death rate	0.10	0.10	0.20
ρ_I	reporting rate for symptomatic infectious	U[0.01, 0.10]	U[0.01, 0.10]	U[0.02, 0.15]
ρ_H	reporting rate for hospitalized people	U[0.95,1]	U[0.95,1]	U[0.95,1]
ρ_{ICU}	reporting rate for ICU admission	0.96	0.96	0.96
ρ_G	reporting rate for hospital discharge	0.96	0.96	0.96
ρ_D	reporting rate for death	0.98	0.98	0.98

*using hospital discharge data and **not using hospital discharge data
***steady-state conditions are defined by: $\frac{dE_1}{dt} = \frac{dE_2}{dt} = \frac{dI_1}{dt} = \frac{dI_2}{dt} = \frac{dA_1}{dt} = \frac{dA_2}{dt} = 0$

S1 Table. (continued)

Parameters	Prior or constant value	Prior or constant value	Prior or constant value	Prior or constant value
	**PACA**	**OC**	**NA**	**ARA**
$I_1(0)$	U[10,500]	U[10,300]	U[10,200]	U[10,1000]
$S(0)$	N=5055000	N=5845000	N=5957000	N=5176000
$E_1(0)$, $E_2(0)$, $I_2(0)$, $A_1(0)$, $A_2(0)$	Use of steady-state conditions ***	Use of steady-state conditions ***	Use of steady-state conditions ***	Use of steady-state conditions ***
$H_1(0)$, $H_2(0)$, $ICU(0)$, $D(0)$, $G(0)$, $R(0)$	0	0	0	0
$\beta(0)$	0.70	0.85	0.70	0.70
ν	U[0.02,0.15]	U[0.02,0.15]	U[0.02,0.15]	U[0.02,0.15]
$1/\sigma$	tN[4,0.1,3,5] (Di Domenico et al, 2020)	tN[4,0.1,3,5] (Di Domenico et al, 2020	tN[4,0.1,3,5] (Di Domenico et al, 2020)	tN[4,0.1,3,5] (Di Domenico et al, 2020)
$1/\gamma$	tN[6,0.2,4.5,7.5] (eg Ferguson et al, 2020)	tN[6,0.2,4.5,7.5] (eg Ferguson et al, 2020)	tN[6,0.2,4.5,7.5] (eg Ferguson et al, 2020)	tN[6,0.2,4.5,7.5] (eg Ferguson et al, 2020)
$1/\kappa$	U[10,20]	U[8,18]	U[8,18]	U[10,20]
$1/\delta$	U[10,20]	U[10,20]	U[14,24]	U[12,22]
τ_A	U[0.30,0.70]	U[0.30,0.70]	U[0.30,0.70]	U[0.30,0.70]
τ_H	U[0.02,0.15]	U[0.02,0.10]	U[0.02,0.10]	U[0.02,0.10]
τ_I	U[0.05,0.15]	U[0.05,0.20]	U[0.05,0.15]	U[0.05,0.15]
τ_D	U[0.10,0.60]	U[0.10,0.60]	U[0.10,0.60]	U[0.10,0.80]
q_1	1.5*q_2 but≤1	1.5*q_2 but≤1	1.5*q_2 but≤1	1.5*q_2 but≤1
q_2	0.55 (Li et al, 2020)	0.55 (Li et al, 2020)	0.55 (Li et al, 2020)	0.55 (Li et al, 2020)
q_I	0.05	0.05	0.05	0.05
q_D	0.10	0.10	0.10	0.10
ρ_I	U[0.02, 0.15]	U[0.02, 0.15]	U[0.02, 0.15]	U[0.01, 0.10]
ρ_H	U[0.95,1]	U[0.95,1]	U[0.95,1]	U[0.95,1]
ρ_{ICU}	0.96	0.96	0.96	0.96
ρ_G	0.96	0.96	0.96	0.96
ρ_D	0.98	0.98	0.98	0.98

S2 Table. Posteriors of the parameters for Ile-de-France region, Ireland, and four French regions: Provence Alpes Côte d'Azur (PACA), Occitanie (OC), Nouvelle-Aquitaine (NA), Auvergne Rhône Alpes (ARA).

**Parameters**	**Definitions**	**Posterior** Median, [95%CI]	**Posterior** Median, [95%CI]	**Posterior** Median, [95%CI]
		**Ile de France***	**Ile de France****	**Ireland**
$I_I(0)$	Initial condition	920, [429-1410]	880, [487-1328]	43, [19–85]
ν	Volatility of the Brownian process	0.104, [0.077-0.129]	0.102, [0.078-0.137]	0.133, [0.101-0.149]
$1/\sigma$	average duration of the incubation period	3.99, [3.81-4.20]	4.00, [3.83-4.15]	3.99, [3.80-4.19]
$1/\gamma$	average duration of the infectious period	6.02, [5.63-6.41]	6.01, [5.65-6.37]	6.01, [5.62-6.35]
$1/\kappa$	average hospitalization period	12.32, [10.58-14.22]	14.91, [13.13-16.65]	13.57, [12.06-15.00]
$1/\delta$	average time spent in ICU	15.61, [13.48-17.96]	15.69, [13.30-17.87]	17.32, [14.99-19.53]
τ_A	fraction of asymptomatics	0.556, [0.313-0.693]	0.535, [0.315-0.692]	0.488, [0.306-0.686]
τ_H	average hospitalization period	0.028, [0.021-0.036]	0.022, [0.021-0.032]	0.027, [0.020-0.044]
τ_I	fraction of ICU admission	0.066, [0.056-0.087]	0.072, [0.062-0.092]	0.030, [0.022-0.044]
τ_D	death rate	0.456, [0.413-0.509]	0.494, [0.452-0.542]	0.411, [0.362-0.460]
ρ_I	reporting rate for symptomatic infectious	0.023, [0.015-0.033]	0.026, [0.016-0.035]	0.092, [0.061-0.143]
ρ_H	reporting rate for hospitalized people	0.976, [0.952-0.998]	0.980, [0.952-0.998]	0.971, [0.951-0.997]

*using hospital discharge data and **not using hospital discharge data

S2 Table. (continued)

**Parameters**	**Posterior** Median, [95%CI]	**Posterior** Median, [95%CI]	**Posterior** Median, [95%CI]	**Posterior** Median, [95%CI]
	**PACA**	**OC**	**NA**	**ARA**
$I_I(0)$	150, [76-249]	101, [44-243]	85, [39-147]	422, [215-815]
ν	0.115, [0.082-0.142]	0.124, [0.094-0.148]	0.133, [0.101-0.144]	0.118, [0.088-0.147]
$1/\sigma$	4.00, [3.82-4.15]	4.01, [3.81-4.21]	3.99, [3.80-4.16]	4.00, [3.81-4.15]
$1/\gamma$	5.97, [5.67-6.28]	5.99, [5.57-6.39]	5.91, [5.65-6.31]	5.99, [5.62-6.35]
$1/\kappa$	14.37, [13.14-15.95]	11.32, [9.92-12.87]	13.81, [12.14-15.51]	14.95, [12.91-17.12]
$1/\delta$	13.85, [12.46-15.35]	13.65, [12.14-15.21]	17.34, [15.27-19.30]	14.77, [12.48-17.48]
τ_A	0.522, [0.322-0.668]	0.514, [0.317-0.690]	0.482, [0.301-0.681]	0. 526, [0.323-0.687]
τ_H	0.023, [0.020-0.031]	0.024, [0.021-0.038]	0.029, [0.021-0.041]	0.023, [0.021-0.036]
τ_I	0.066, [0.055-0.082]	0.075, [0.063-0.103]	0.070, [0.054-0.092]	0.061, [0.052-0.082]
τ_D	0.412, [0.366-0.456]	0.350, [0.305-0.392]	0.433, [0.379-0.483]	0.464, [0.409-0.521]
ρ_I	0.048, [0.035-0.069]	0.026, [0.021-0.045]	0.032, [0.022-0.051]	0.022, [0.014-0.033]
ρ_H	0.987, [0.956-0.999]	0.980, [0.953-0.998]	0.980, [0.955-0.995]	0.976, [0.951-0.998]
