# Supplementary material for: A mechanistic and data-driven reconstruction of the time-varying reproduction number: Application to the COVID-19 epidemic
Source: PLoS Comput Biol. 2021 Jul 26;17(7):e1009211. doi: 10.1371/journal.pcbi.1009211 (PMC8341713; doi:10.1371/journal.pcbi.1009211)
Supplement: S1 Table — (PDF) [file pcbi.1009211.s002.pdf]

S1 Table. Definition of the different parameters and their priors for Ile-de-France region, Ireland and four other French regions: Provence Alpes Côte d'Azur (PACA), Occitanie (OC), Nouvelle-Aquitaine (NA), Auvergne Rhône Alpes (ARA). The priors have been chosen based on current literature.
U stands for uniform distribution and tN for truncated normal distribution (tN[mean,std,limit inf,limit sup]).

**Parameters**	**Definitions**	**Prior or constant value**	**Prior or constant value**	**Prior or constant value**
		**Ile de France***	**Ile de France****	**Ireland**
$I_1(0)$	Initial condition	U[10,1500]	U[10,1500]	U[5,100]
$S(0)$	Initial condition	N=12278000	N=12278000	N=5176000
$E_1(0)$, $E_2(0)$, $I_2(0)$, $A_1(0)$, $A_2(0)$	Initial conditions	Use of steady-state conditions ***	Use of steady-state conditions ***	Use of steady-state conditions ***
$H_1(0)$, $H_2(0)$, $ICU(0)$, $D(0)$, $G(0)$, $R(0)$	Initials conditions	0	0	0
$\beta(0)$	Initial condition of the transmission rate	0.85	0.85	0.70
ν	Volatility of the Brownian process	U[0.02,0.15]	U[0.02,0.15]	U[0.05,0.15]
$1/\sigma$	average duration of the incubation period	tN[4,0.1,3,5] (Di Domenico et al, 2020)	tN[4,0.1,3,5] (Di Domenico et al, 2020)	tN[4,0.1,3,5] (Di Domenico et al, 2020)
$1/\gamma$	average duration of the infectious period	tN[6,0.2,4.5,7.5] (eg Ferguson et al, 2020)	tN[6,0.2,4.5,7.5] (eg Ferguson et al, 2020)	tN[6,0.2,4.5,7.5] (eg Ferguson et al, 2020)
$1/\kappa$	average hospitalization period	U[10,20]	U[10,20]	U[8,20]
$1/\delta$	average time spent in ICU	U[10,20]	U[10,20]	U[8,20]
τ_A	fraction of asymptomatics	U[0.30,0.70]	U[0.30,0.70]	U[0.30,0.70]
τ_H	fraction of hospitalization	U[0.02,0.10]	U[0.02,0.10]	U[0.02,0.10]
τ_I	fraction of ICU admission	U[0.05,0.15]	U[0.05,0.15]	U[0.025,0.15]
τ_D	death rate	U[0.10,0.80]	U[0.10,0.80]	U[0.10,0.60]
q_1	reduction in transmissibility	$1.5*q_2$ but≤ 1	$1.5*q_2$ but≤ 1	$1.5*q_2$ but≤ 1
q_2	reduction in transmissibility	0.55 (Li et al, 2020)	0.55 (Li et al, 2020)	0.55 (Li et al, 2020)
q_I	reduction in ICU admission fraction	0.05	0.05	0.10
q_D	reduction in death rate	0.10	0.10	0.20
ρ_I	reporting rate for symptomatic infectious	U[0.01, 0.10]	U[0.01, 0.10]	U[0.02, 0.15]
ρ_H	reporting rate for hospitalized people	U[0.95,1]	U[0.95,1]	U[0.95,1]
ρ_{ICU}	reporting rate for ICU admission	0.96	0.96	0.96
ρ_G	reporting rate for hospital discharge	0.96	0.96	0.96
ρ_D	reporting rate for death	0.98	0.98	0.98

*using hospital discharge data and **not using hospital discharge data
***steady-state conditions are defined by: $\frac{dE_1}{dt}=\frac{dE_2}{dt}=\frac{dI_1}{dt}=\frac{dI_2}{dt}=\frac{dA_1}{dt}=\frac{dA_2}{dt}=0$

S1 Table. (continued)

Parameters	Prior or constant value	Prior or constant value	Prior or constant value	Prior or constant value
	**PACA**	**OC**	**NA**	**ARA**
$I_1(0)$	U[10,500]	U[10,300]	U[10,200]	U[10,1000]
$S(0)$	N=5055000	N=5845000	N=5957000	N=5176000
$E_1(0)$, $E_2(0)$, $I_2(0)$, $A_1(0)$, $A_2(0)$	Use of steady-state conditions ***	Use of steady-state conditions ***	Use of steady-state conditions ***	Use of steady-state conditions ***
$H_1(0)$, $H_2(0)$, $ICU(0)$, $D(0)$, $G(0)$, $R(0)$	0	0	0	0
$\beta(0)$	0.70	0.85	0.70	0.70
ν	U[0.02,0.15]	U[0.02,0.15]	U[0.02,0.15]	U[0.02,0.15]
$1/\sigma$	tN[4,0.1,3,5] (Di Domenico et al, 2020)	tN[4,0.1,3,5] (Di Domenico et al, 2020	tN[4,0.1,3,5] (Di Domenico et al, 2020)	tN[4,0.1,3,5] (Di Domenico et al, 2020)
$1/\gamma$	tN[6,0.2,4.5,7.5] (eg Ferguson et al, 2020)	tN[6,0.2,4.5,7.5] (eg Ferguson et al, 2020)	tN[6,0.2,4.5,7.5] (eg Ferguson et al, 2020)	tN[6,0.2,4.5,7.5] (eg Ferguson et al, 2020)
$1/\kappa$	U[10,20]	U[8,18]	U[8,18]	U[10,20]
$1/\delta$	U[10,20]	U[10,20]	U[14,24]	U[12,22]
τ_A	U[0.30,0.70]	U[0.30,0.70]	U[0.30,0.70]	U[0.30,0.70]
τ_H	U[0.02,0.15]	U[0.02,0.10]	U[0.02,0.10]	U[0.02,0.10]
τ_I	U[0.05,0.15]	U[0.05,0.20]	U[0.05,0.15]	U[0.05,0.15]
τ_D	U[0.10,0.60]	U[0.10,0.60]	U[0.10,0.60]	U[0.10,0.80]
q_1	1.5*q_2 but≤1	1.5*q_2 but≤1	1.5*q_2 but≤1	1.5*q_2 but≤1
q_2	0.55 (Li et al, 2020)	0.55 (Li et al, 2020)	0.55 (Li et al, 2020)	0.55 (Li et al, 2020)
q_I	0.05	0.05	0.05	0.05
q_D	0.10	0.10	0.10	0.10
ρ_I	U[0.02, 0.15]	U[0.02, 0.15]	U[0.02, 0.15]	U[0.01, 0.10]
ρ_H	U[0.95,1]	U[0.95,1]	U[0.95,1]	U[0.95,1]
ρ_{ICU}	0.96	0.96	0.96	0.96
ρ_G	0.96	0.96	0.96	0.96
ρ_D	0.98	0.98	0.98	0.98
