# Supplementary material for: A mechanistic and data-driven reconstruction of the time-varying reproduction number: Application to the COVID-19 epidemic
Source: PLoS Comput Biol. 2021 Jul 26;17(7):e1009211. doi: 10.1371/journal.pcbi.1009211 (PMC8341713; doi:10.1371/journal.pcbi.1009211)
Supplement: S2 Table — (PDF) [file pcbi.1009211.s003.pdf]

S2 Table. Posteriors of the parameters for Ile-de-France region, Ireland, and four French regions: Provence Alpes Côte d'Azur (PACA), Occitanie (OC), Nouvelle-Aquitaine (NA), Auvergne Rhône Alpes (ARA).

**Parameters**	**Definitions**	**Posterior** Median, [95%CI]	**Posterior** Median, [95%CI]	**Posterior** Median, [95%CI]
		**Ile de France***	**Ile de France****	**Ireland**
$I_I(0)$	Initial condition	920, [429-1410]	880, [487-1328]	43, [19–85]
ν	Volatility of the Brownian process	0.104, [0.077-0.129]	0.102, [0.078-0.137]	0.133, [0.101-0.149]
$1/\sigma$	average duration of the incubation period	3.99, [3.81-4.20]	4.00, [3.83-4.15]	3.99, [3.80-4.19]
$1/\gamma$	average duration of the infectious period	6.02, [5.63-6.41]	6.01, [5.65-6.37]	6.01, [5.62-6.35]
$1/\kappa$	average hospitalizion period	12.32, [10.58-14.22]	14.91, [13.13-16.65]	13.57, [12.06-15.00]
$1/\delta$	average time spent in ICU	15.61, [13.48-17.96]	15.69, [13.30-17.87]	17.32, [14.99-19.53]
τ_A	fraction of asymptomatics	0.556, [0.313-0.693]	0.535, [0.315-0.692]	0.488, [0.306-0.686]
τ_H	average hospitalization period	0.028, [0.021-0.036]	0.022, [0.021-0.032]	0.027, [0.020-0.044]
τ_I	fraction of ICU admission	0.066, [0.056-0.087]	0.072, [0.062-0.092]	0.030, [0.022-0.044]
τ_D	death rate	0.456, [0.413-0.509]	0.494, [0.452-0.542]	0.411, [0.362-0.460]
ρ_I	reporting rate for symptomatic infectious	0.023, [0.015-0.033]	0.026, [0.016-0.035]	0.092, [0.061-0.143]
ρ_H	reporting rate for hospitalized people	0.976, [0.952-0.998]	0.980, [0.952-0.998]	0.971, [0.951-0.997]

*using hospital discharge data and **not using hospital discharge data

S2 Table. (continued)

**Parameters**	**Posterior** Median, [95%CI]	**Posterior** Median, [95%CI]	**Posterior** Median, [95%CI]	**Posterior** Median, [95%CI]
	**PACA**	**OC**	**NA**	**ARA**
$I_I(0)$	150, [76-249]	101, [44-243]	85, [39-147]	422, [215-815]
ν	0.115, [0.082-0.142]	0.124, [0.094-0.148]	0.133, [0.101-0.144]	0.118, [0.088-0.147]
$1/\sigma$	4.00, [3.82-4.15]	4.01, [3.81-4.21]	3.99, [3.80-4.16]	4.00, [3.81-4.15]
$1/\gamma$	5.97, [5.67-6.28]	5.99, [5.57-6.39]	5.91, [5.65-6.31]	5.99, [5.62-6.35]
$1/\kappa$	14.37, [13.14-15.95]	11.32, [9.92-12.87]	13.81, [12.14-15.51]	14.95, [12.91-17.12]
$1/\delta$	13.85, [12.46-15.35]	13.65, [12.14-15.21]	17.34, [15.27-19.30]	14.77, [12.48-17.48]
τ_A	0.522, [0.322-0.668]	0.514, [0.317-0.690]	0.482, [0.301-0.681]	0. 526, [0.323-0.687]
τ_H	0.023, [0.020-0.031]	0.024, [0.021-0.038]	0.029, [0.021-0.041]	0.023, [0.021-0.036]
τ_I	0.066, [0.055-0.082]	0.075, [0.063-0.103]	0.070, [0.054-0.092]	0.061, [0.052-0.082]
τ_D	0.412, [0.366-0.456]	0.350, [0.305-0.392]	0.433, [0.379-0.483]	0.464, [0.409-0.521]
ρ_I	0.048, [0.035-0.069]	0.026, [0.021-0.045]	0.032, [0.022-0.051]	0.022, [0.014-0.033]
ρ_H	0.987, [0.956-0.999]	0.980, [0.953-0.998]	0.980, [0.955-0.995]	0.976, [0.951-0.998]
