# Supplementary figures and images for: A mechanistic and data-driven reconstruction of the time-varying reproduction number: Application to the COVID-19 epidemic
Source: PLoS Comput Biol. 2021 Jul 26;17(7):e1009211. doi: 10.1371/journal.pcbi.1009211 (PMC8341713; doi:10.1371/journal.pcbi.1009211)

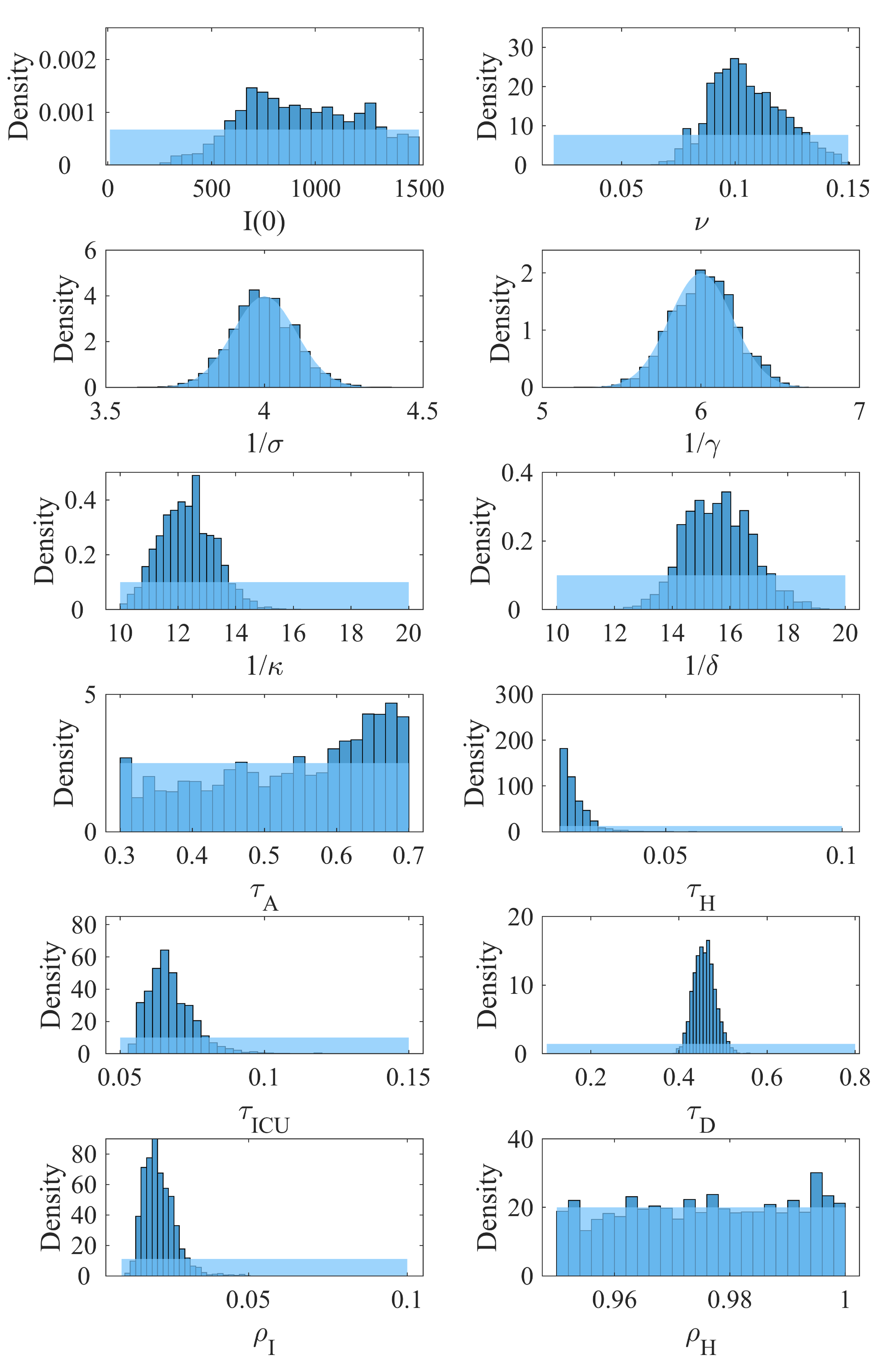

Supplement: S1 Fig — I1(0) is the initial number of infectious individuals, ν is the volatility of the Brownian process of β(t), 1/σ the average duration of the incubation, 1/γ the average duration of infectious period, 1/κ the average hospitalization period, 1/δ the average time spent in ICU, τA the fraction of asymptomatics, τH the fraction of infectious hospitalized, τI the fraction of ICU admission, τD the death rate, ρI the reporting rate for the infectious, ρH the reporting rate for the hospitalized people. (TIF) [file pcbi.1009211.s004.tif]

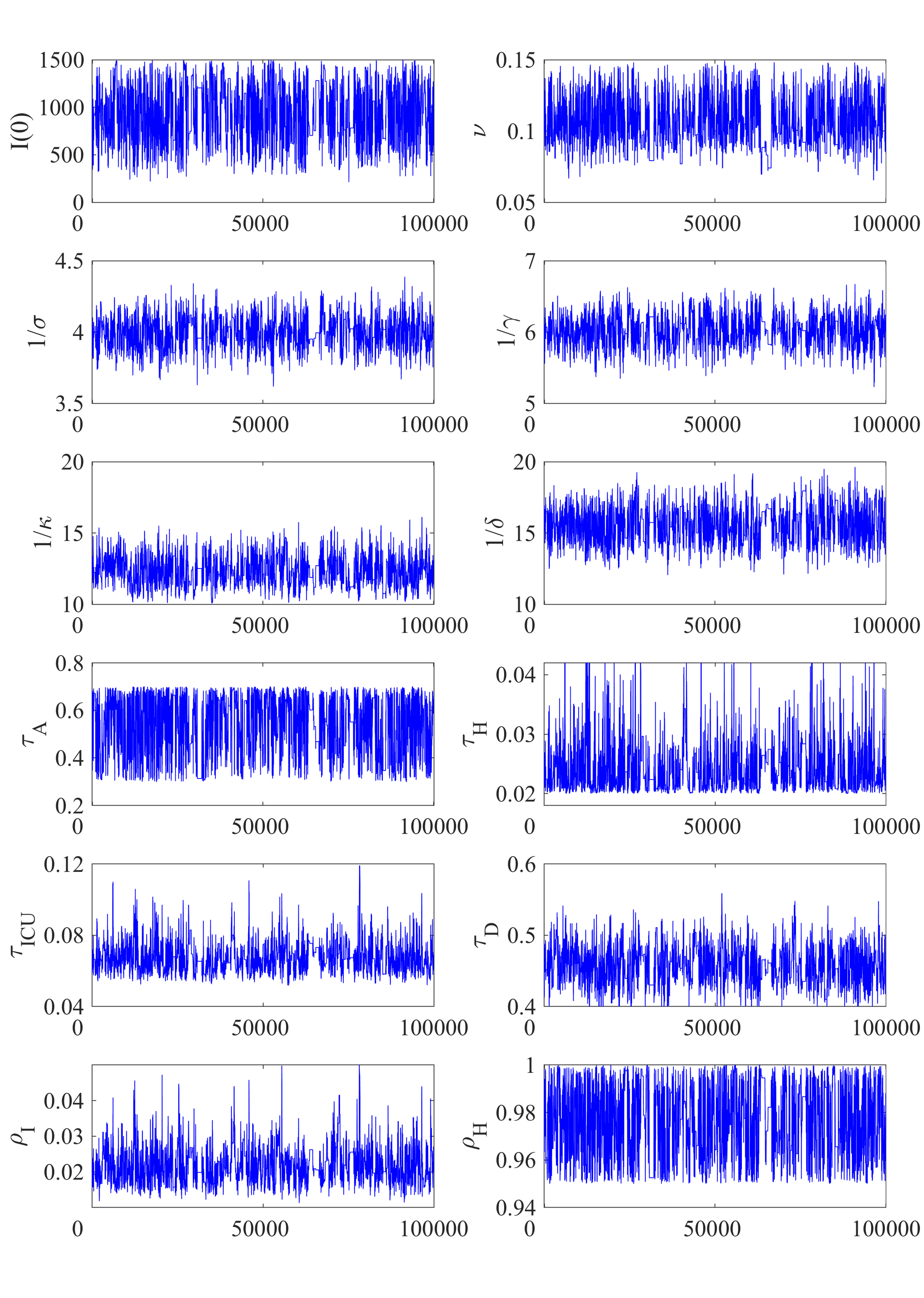

Supplement: S2 Fig — I1(0) is the initial number of infectious, ν is the volatility of the Brownian process of β(t), 1/σ the average duration of the incubation, 1/γ the average duration of infectious period, 1/κ the average hospitalization period, 1/δ the average time spent in ICU, τA the fraction of asymptomatics, τH the fraction of infectious hospitalized, τI the fraction of ICU admission, τD the death rate, ρI the reporting rate for the infectious, ρH the reporting rate for the hospitalized people. The acceptance rate is equal to 13.6% and the chain is stationary, ie stationarity is not rejected at the 5% level by the Geweke diagnosis. (TIF) [file pcbi.1009211.s005.tif]

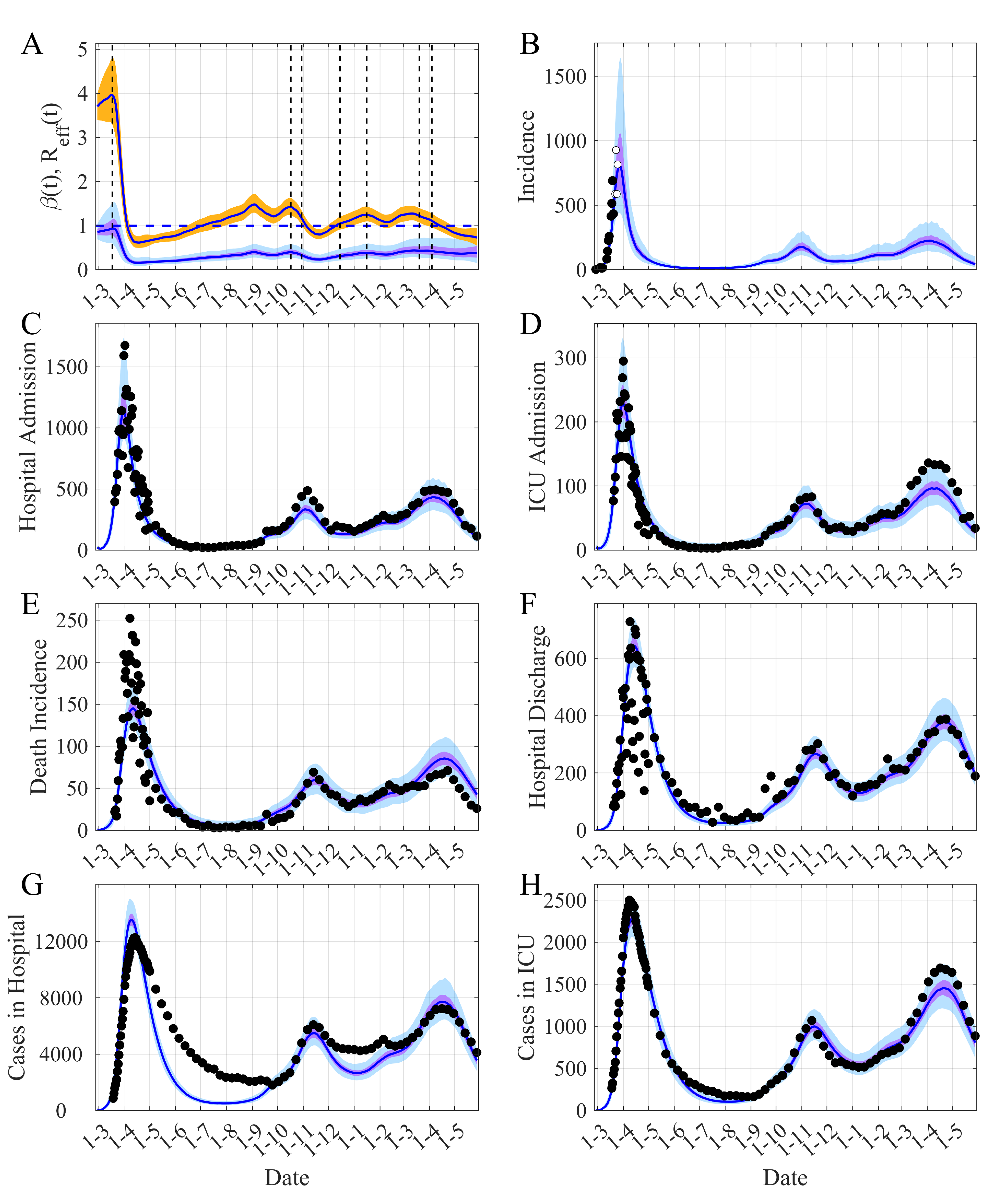

Supplement: S3 Fig — Caption as for Fig 2. The black points are observations used by the inference process, the white points are the observations not used. (TIF) [file pcbi.1009211.s006.tif]

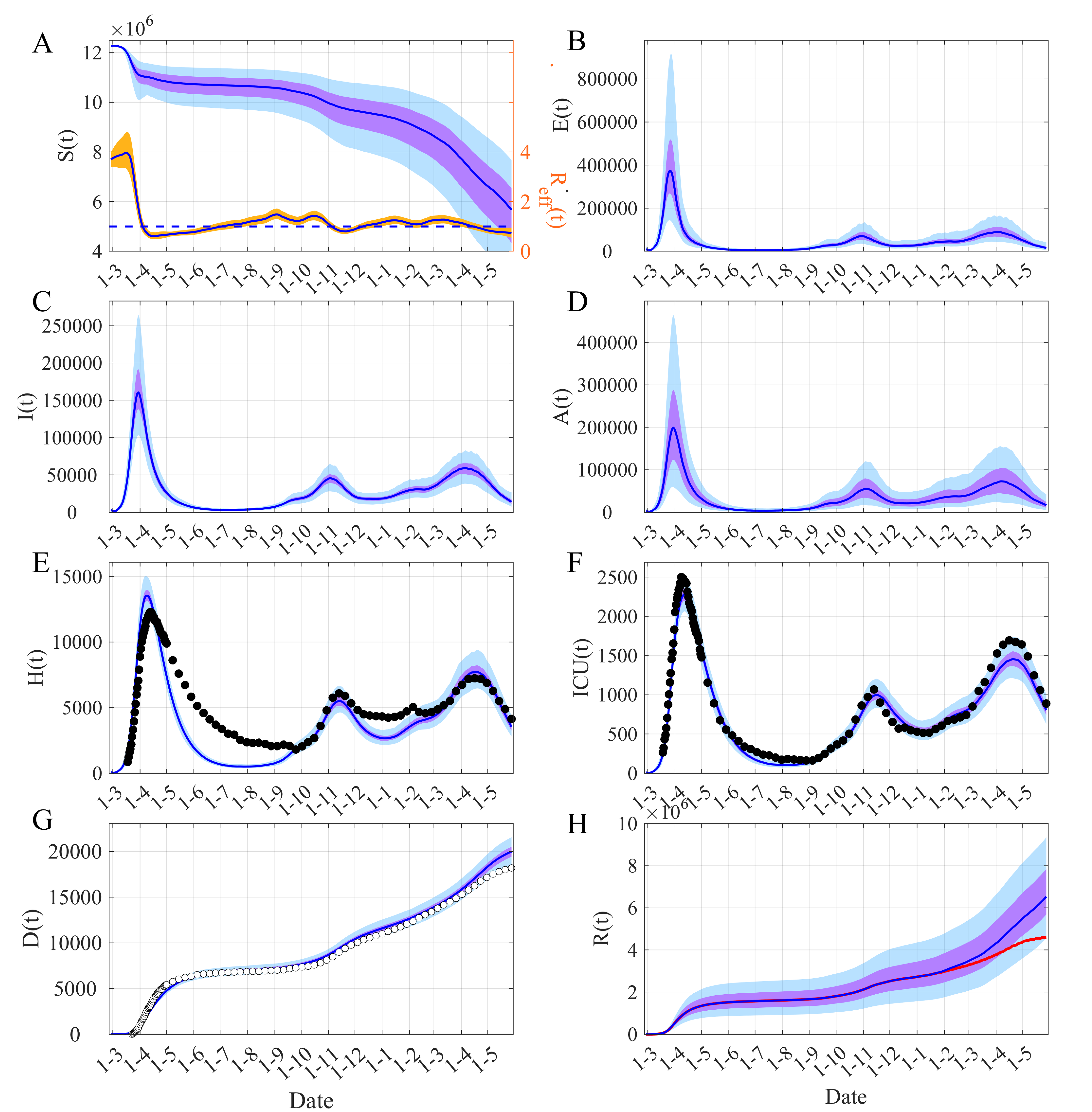

Supplement: S4 Fig — The black points are observations used by the inference process, the white points are the observations not used. (TIF) [file pcbi.1009211.s007.tif]

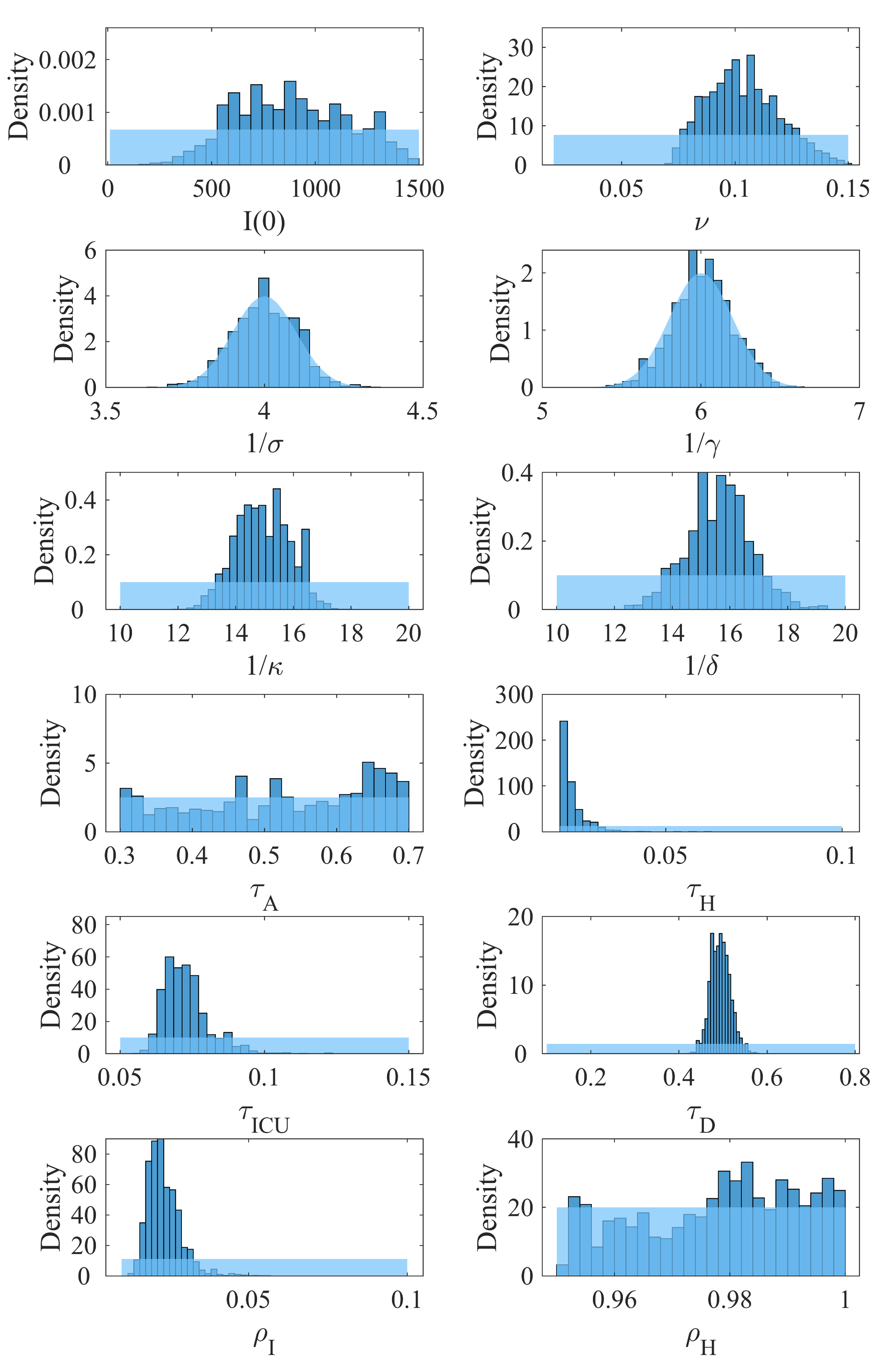

Supplement: S5 Fig — Caption as for S1 Fig. (TIF) [file pcbi.1009211.s008.tif]

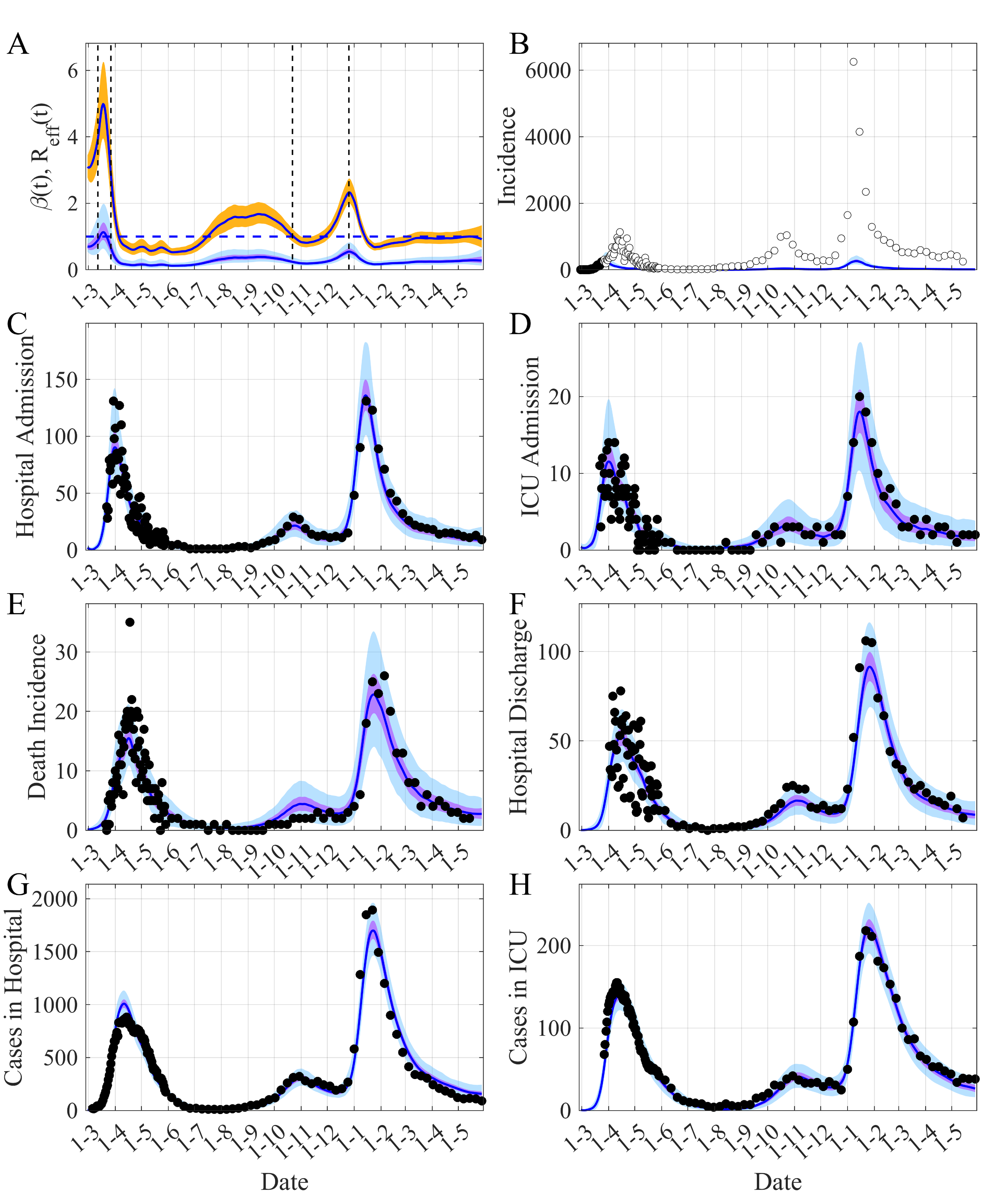

Supplement: S6 Fig — Caption as for Fig 2 but average daily data of the current week is used after 01-06-2020. The black points are observations used by the inference process, the white points are the observations not used. (TIF) [file pcbi.1009211.s009.tif]

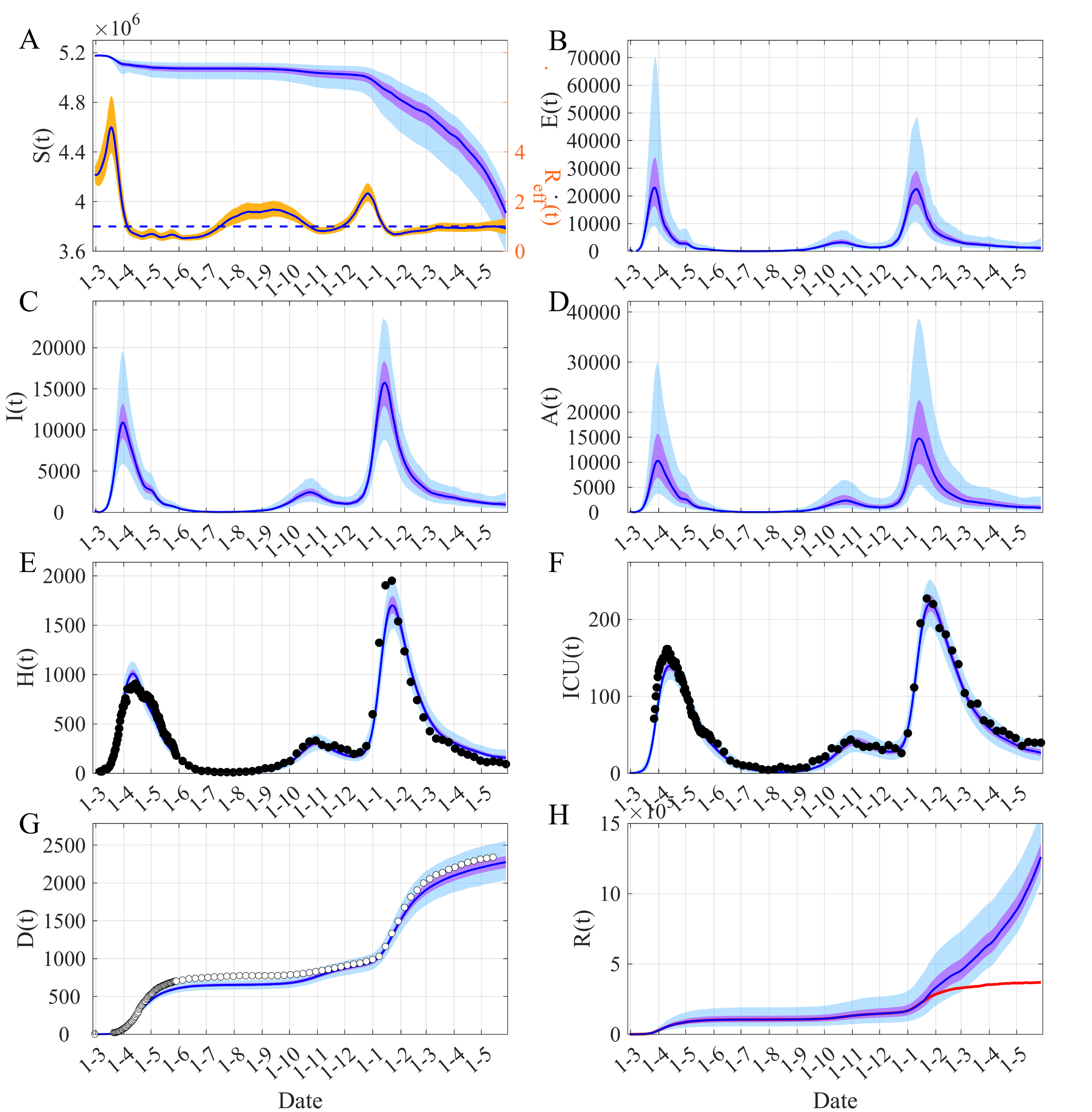

Supplement: S7 Fig — Caption as for Fig 3. The black points are observations used by the inference process, the white points are the observations not used. (TIF) [file pcbi.1009211.s010.tif]

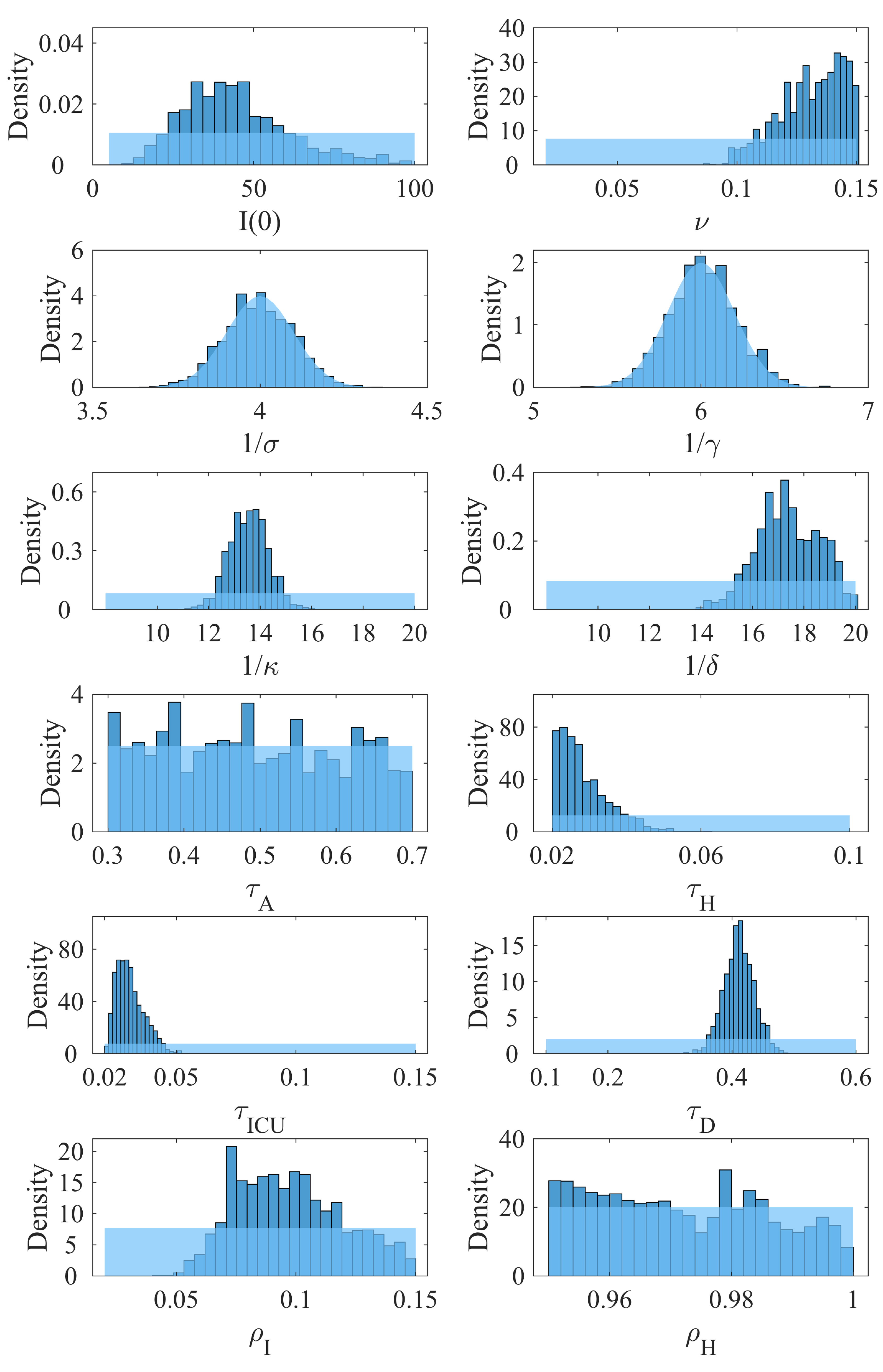

Supplement: S8 Fig — Caption as for S1 Fig. (TIF) [file pcbi.1009211.s011.tif]

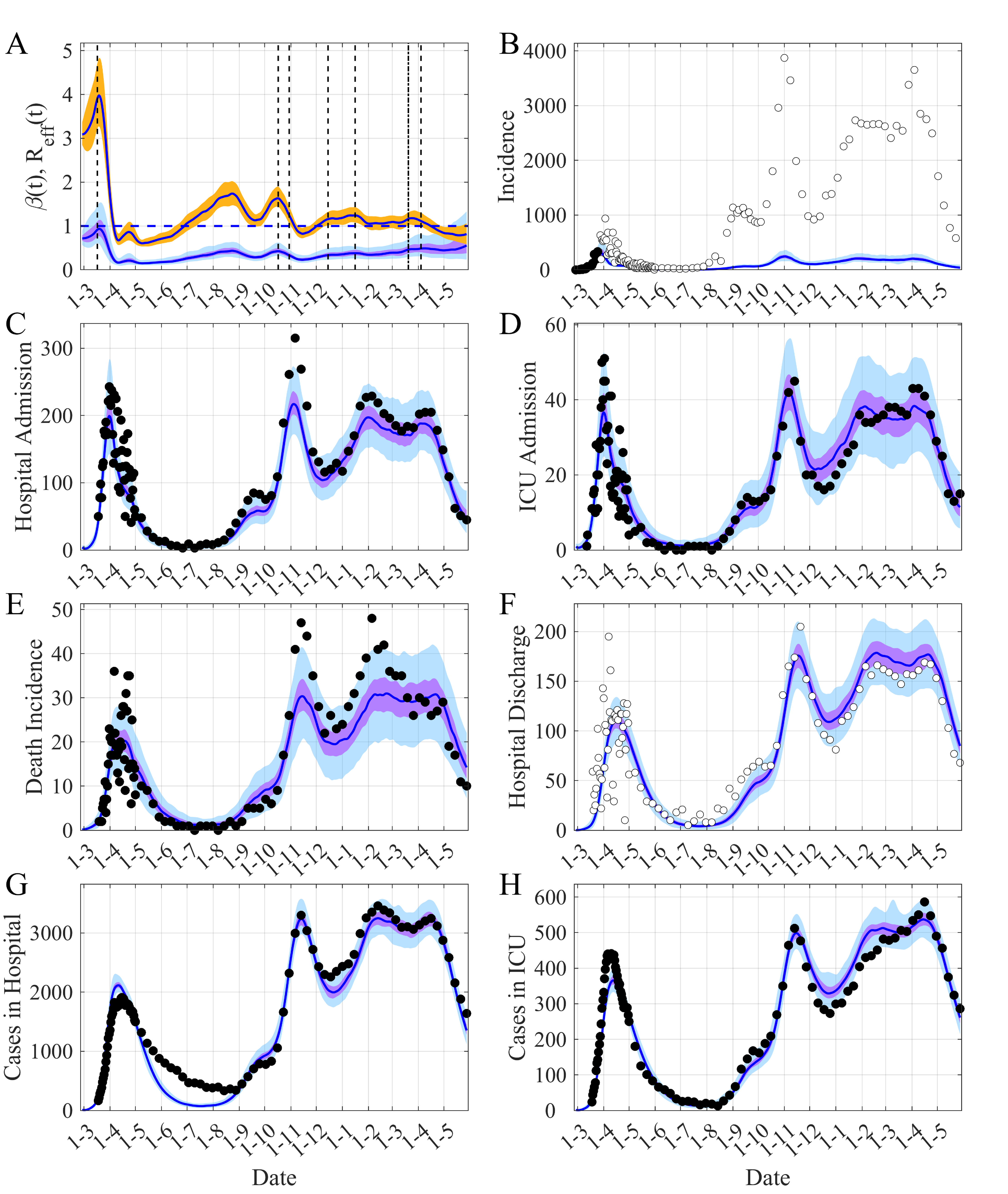

Supplement: S9 Fig — Caption as for Fig 2. The black points are observations used by the inference process, the white points are the observations not used. (TIF) [file pcbi.1009211.s012.tif]

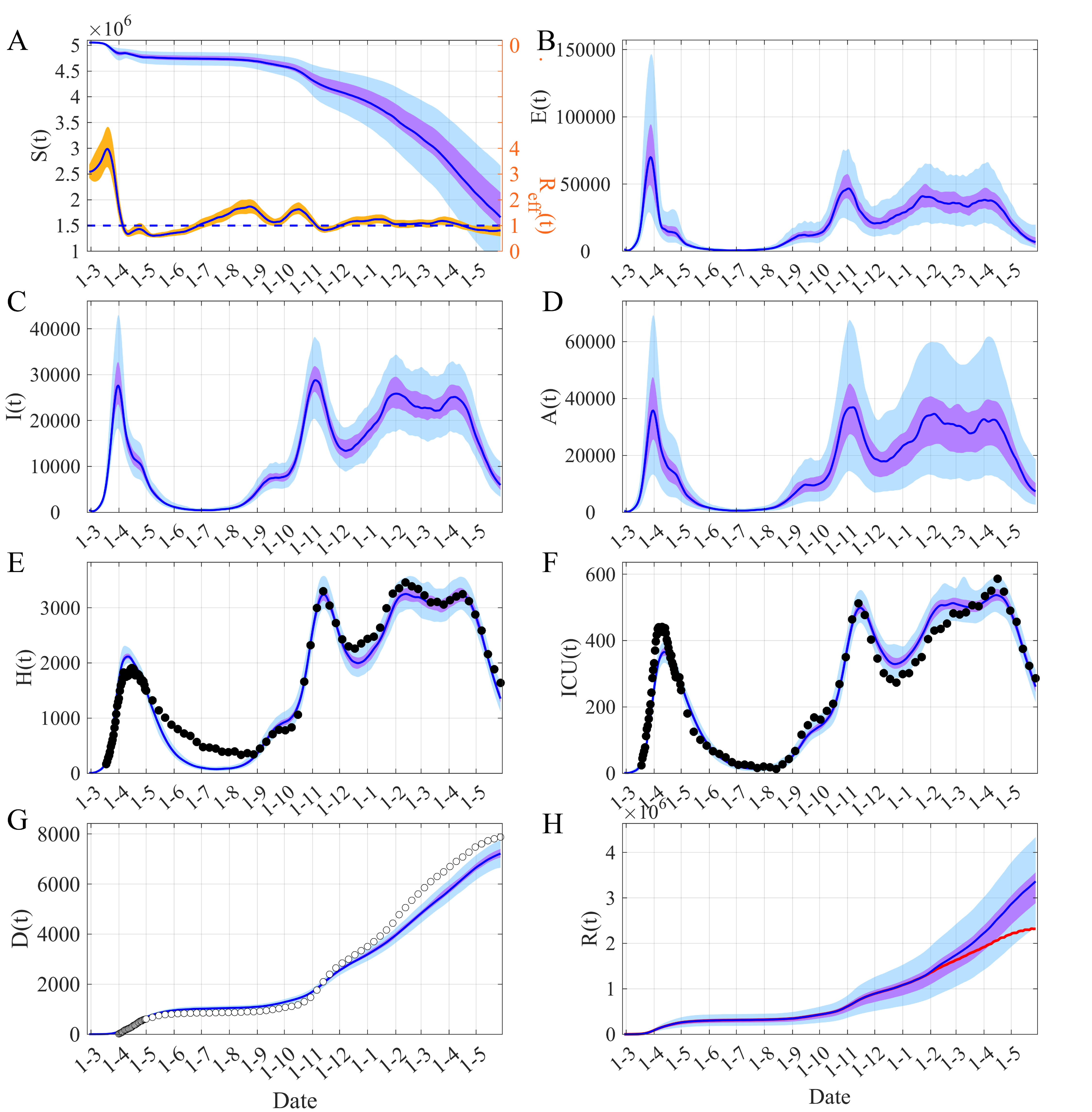

Supplement: S10 Fig — Caption as for Fig 3. The black points are observations used by the inference process, the white points are the observations not used. (TIF) [file pcbi.1009211.s013.tif]

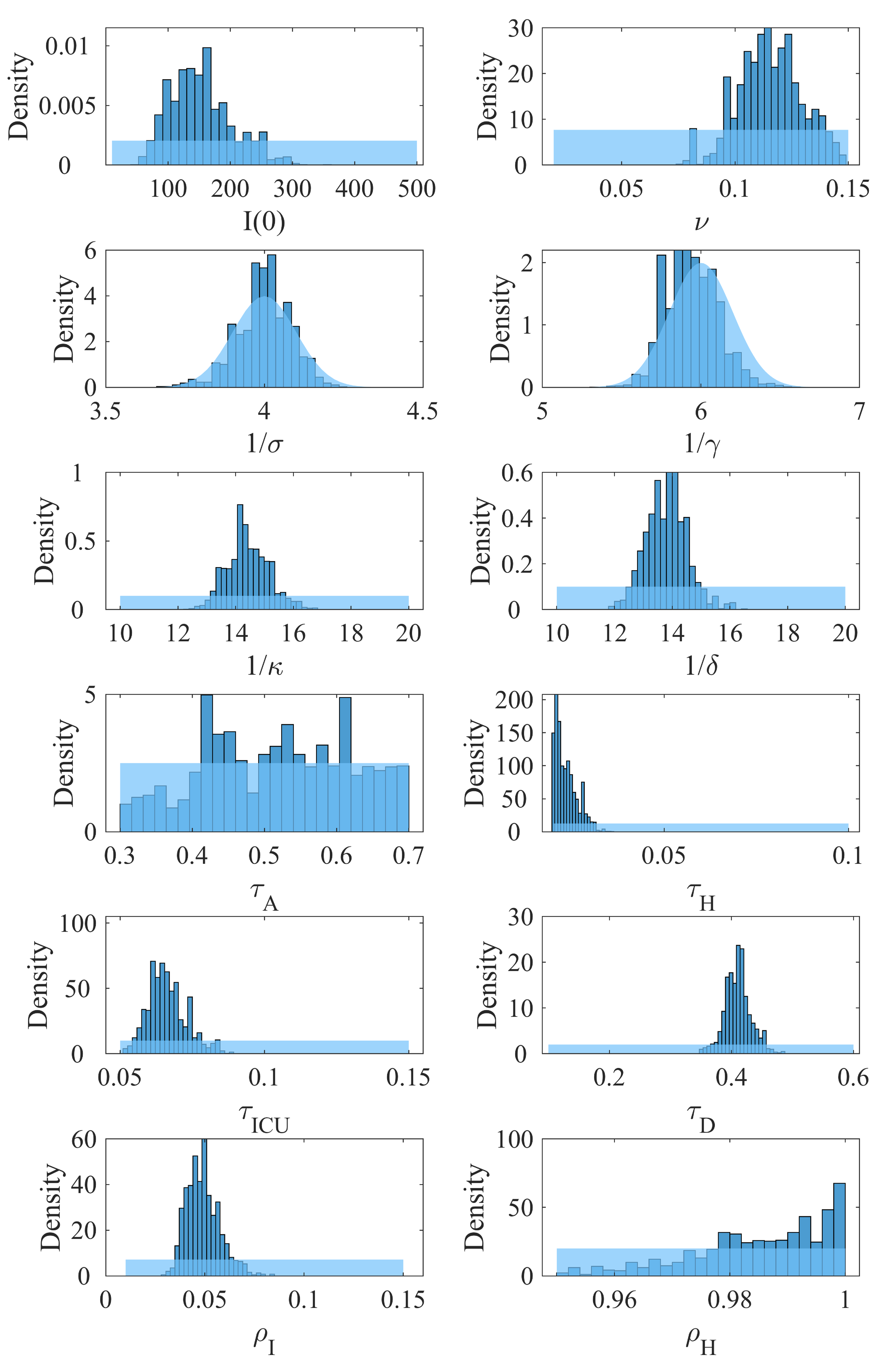

Supplement: S11 Fig — Caption as for S1 Fig. (TIF) [file pcbi.1009211.s014.tif]

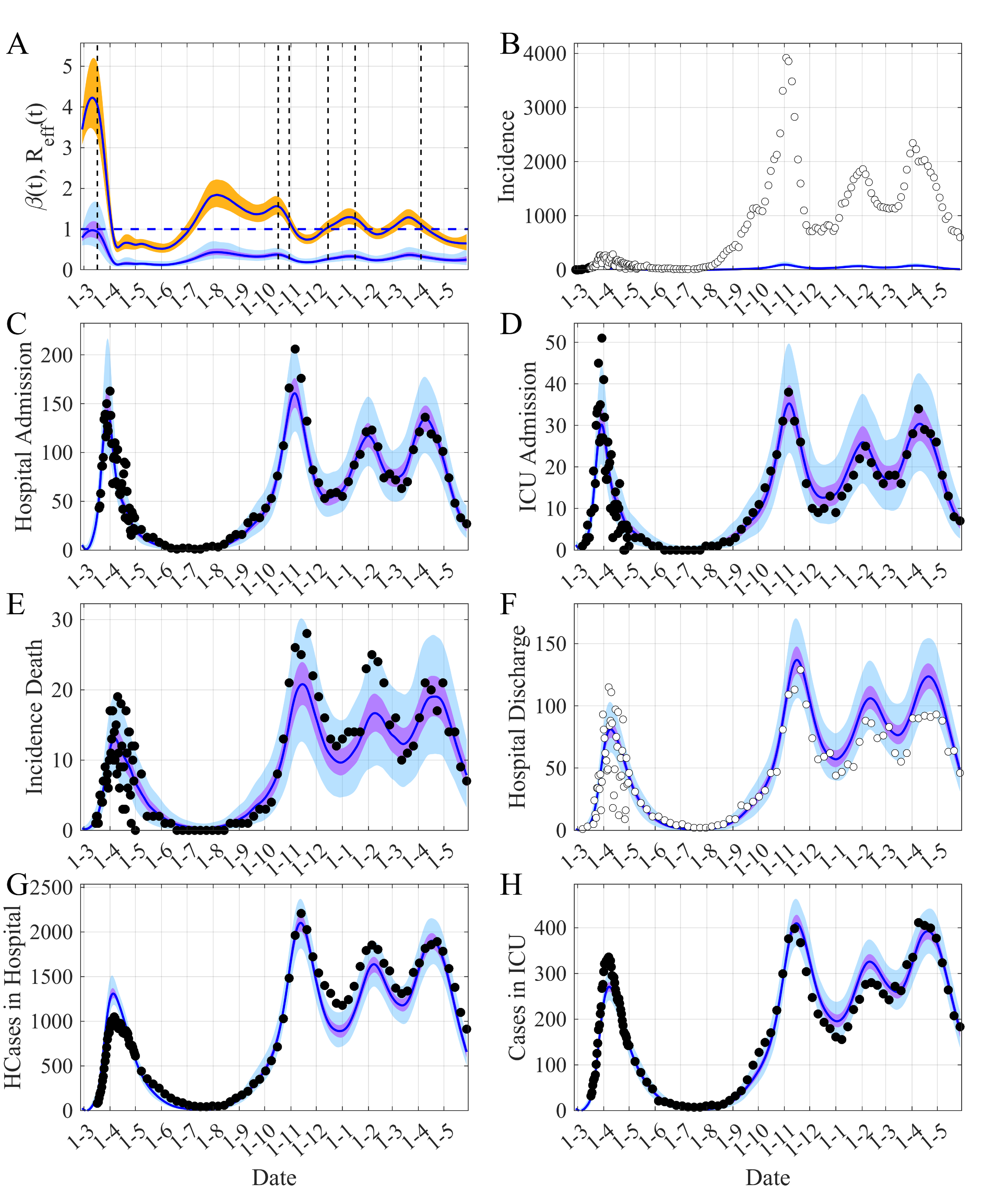

Supplement: S12 Fig — Caption as for Fig 2. The black points are observations used by the inference process, the white points are the observations not used. (TIF) [file pcbi.1009211.s015.tif]

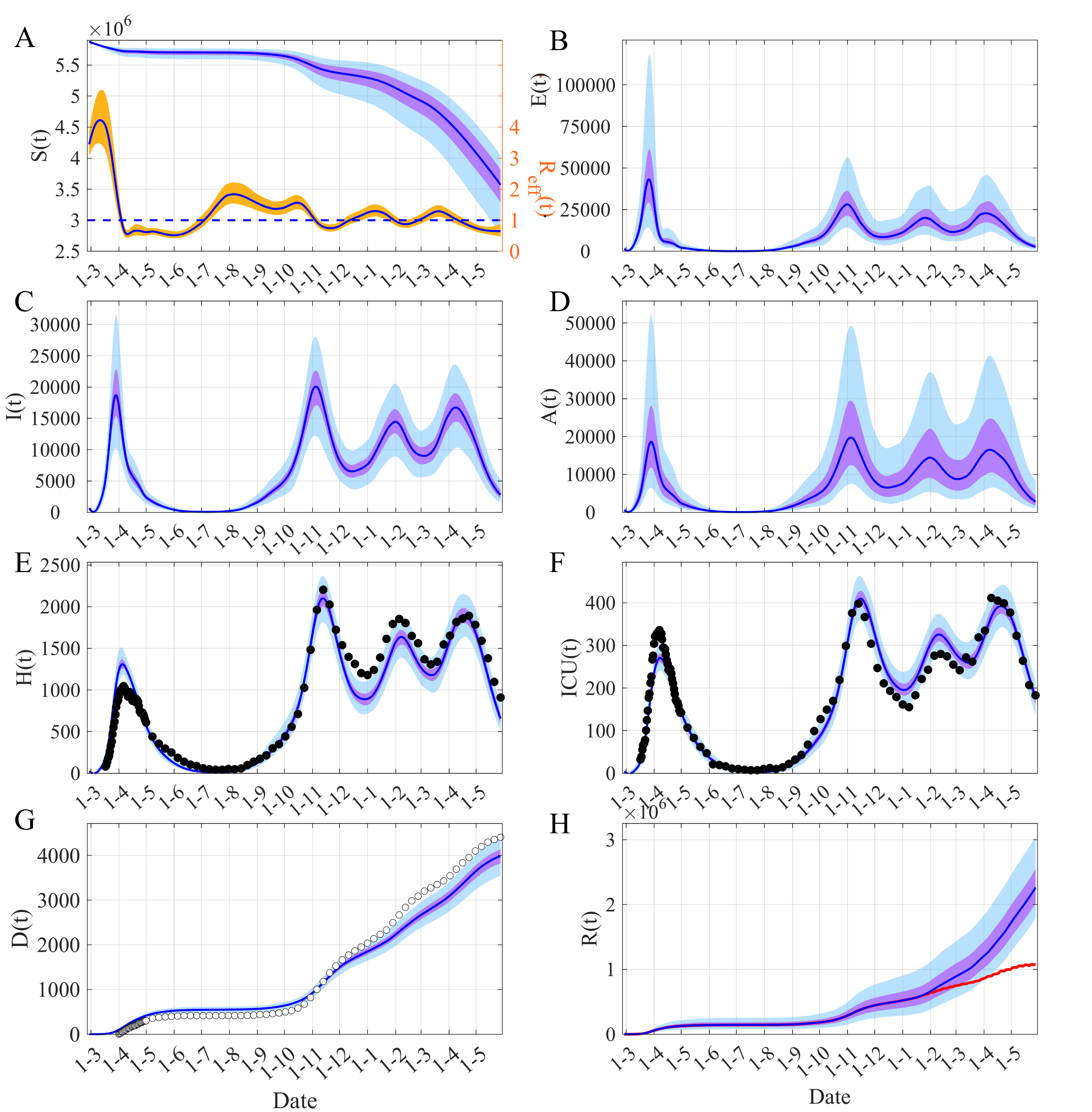

Supplement: S13 Fig — The black points are observations used by the inference process, the white points are the observations not used. (TIF) [file pcbi.1009211.s016.tif]

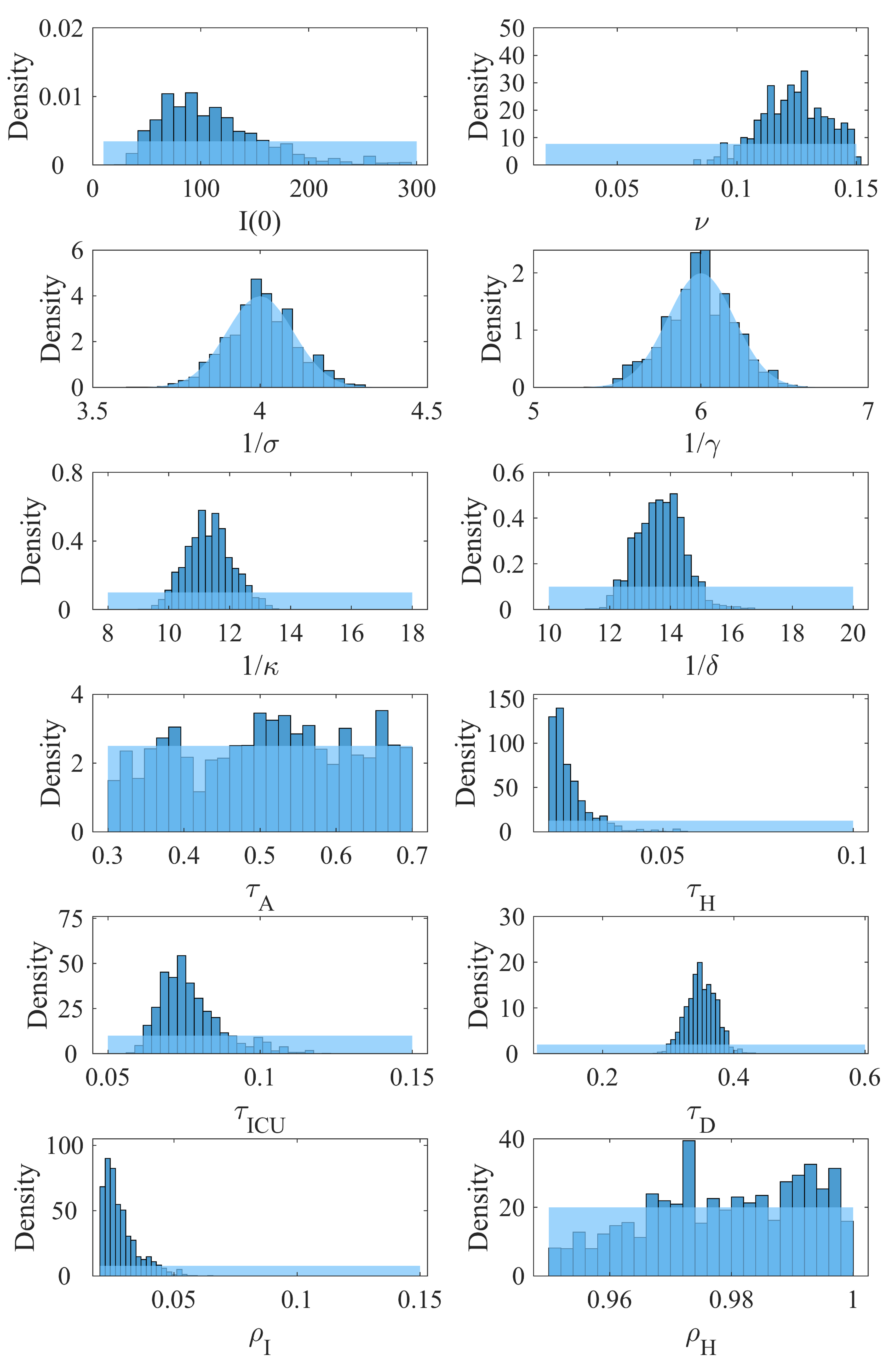

Supplement: S14 Fig — Caption as for S1 Fig. (TIF) [file pcbi.1009211.s017.tif]

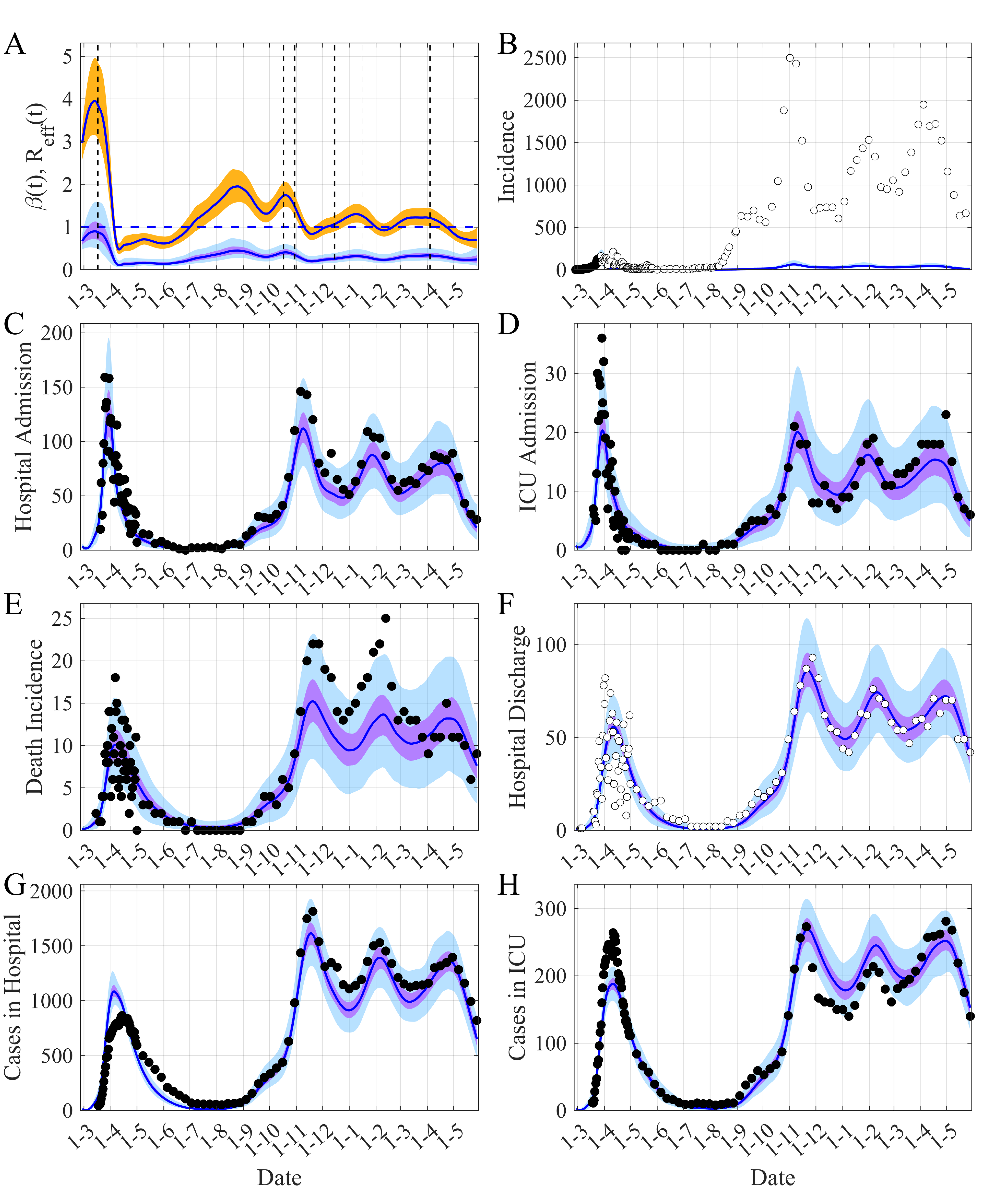

Supplement: S15 Fig — Caption as for Fig 2. The black points are observations used by the inference process, the white points are the observations not used. (TIF) [file pcbi.1009211.s018.tif]

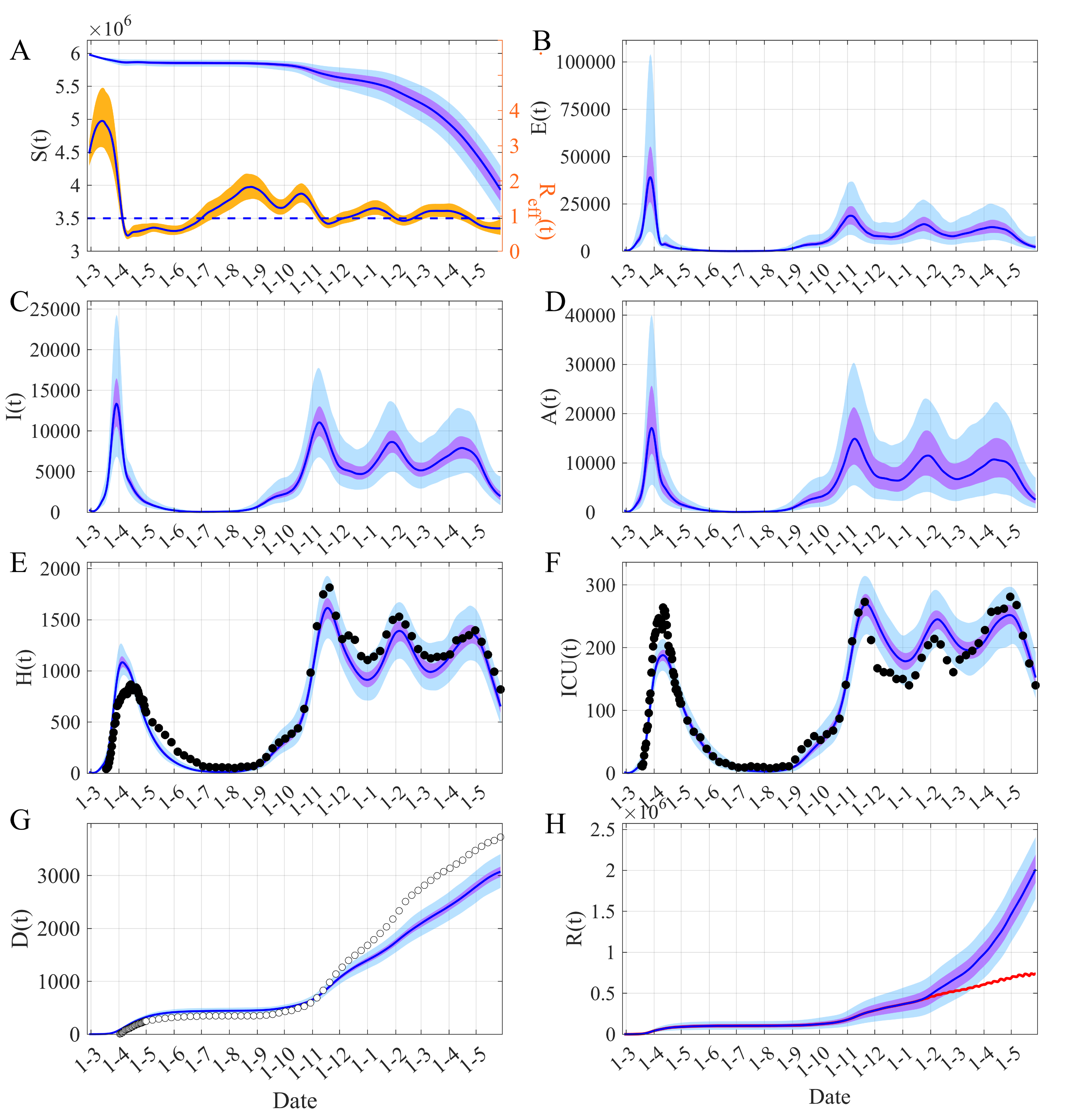

Supplement: S16 Fig — Caption as for Fig 3. The black points are observations used by the inference process, the white points are the observations not used. (TIF) [file pcbi.1009211.s019.tif]

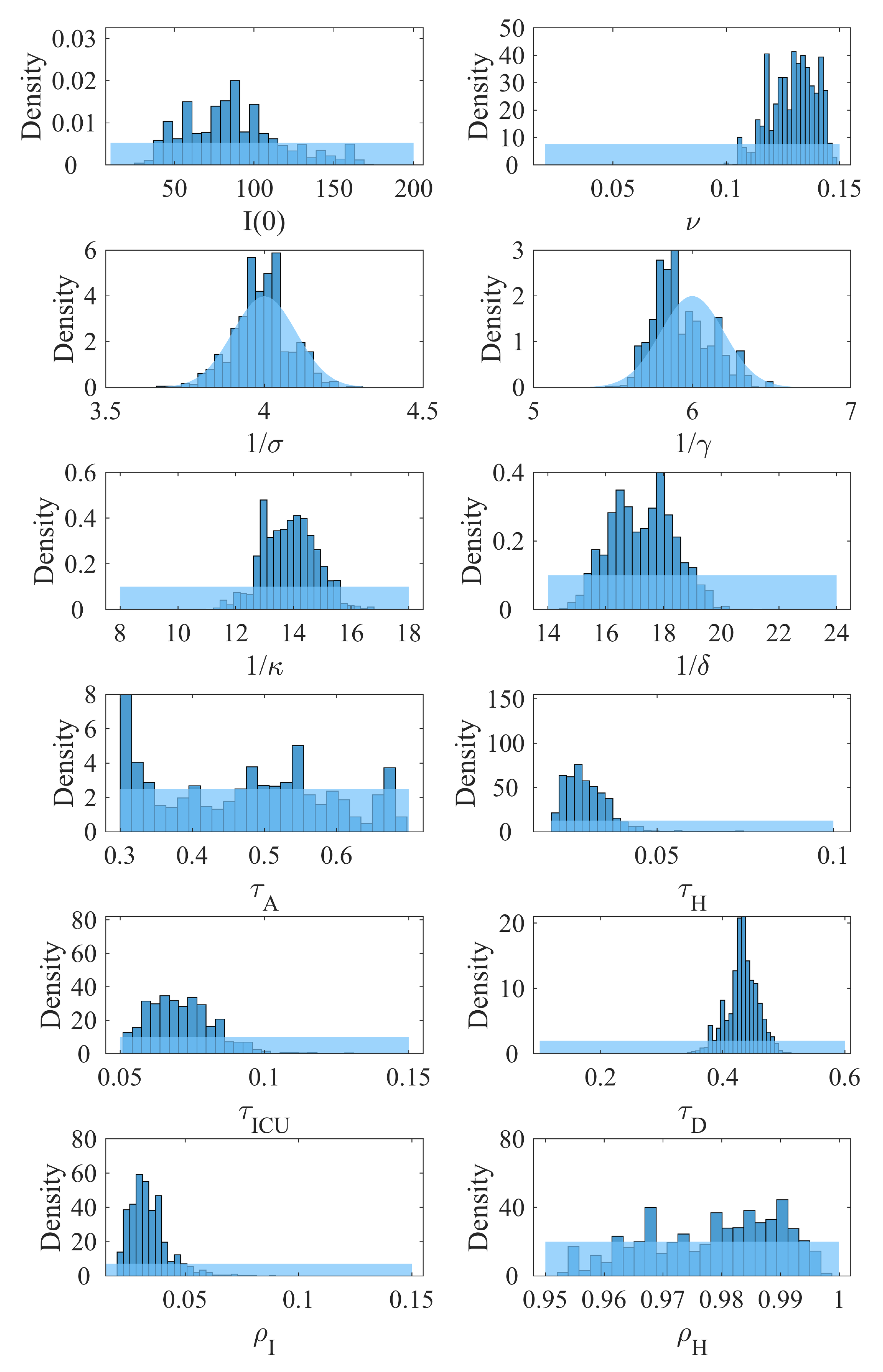

Supplement: S17 Fig — Caption as for S1 Fig. (TIF) [file pcbi.1009211.s020.tif]

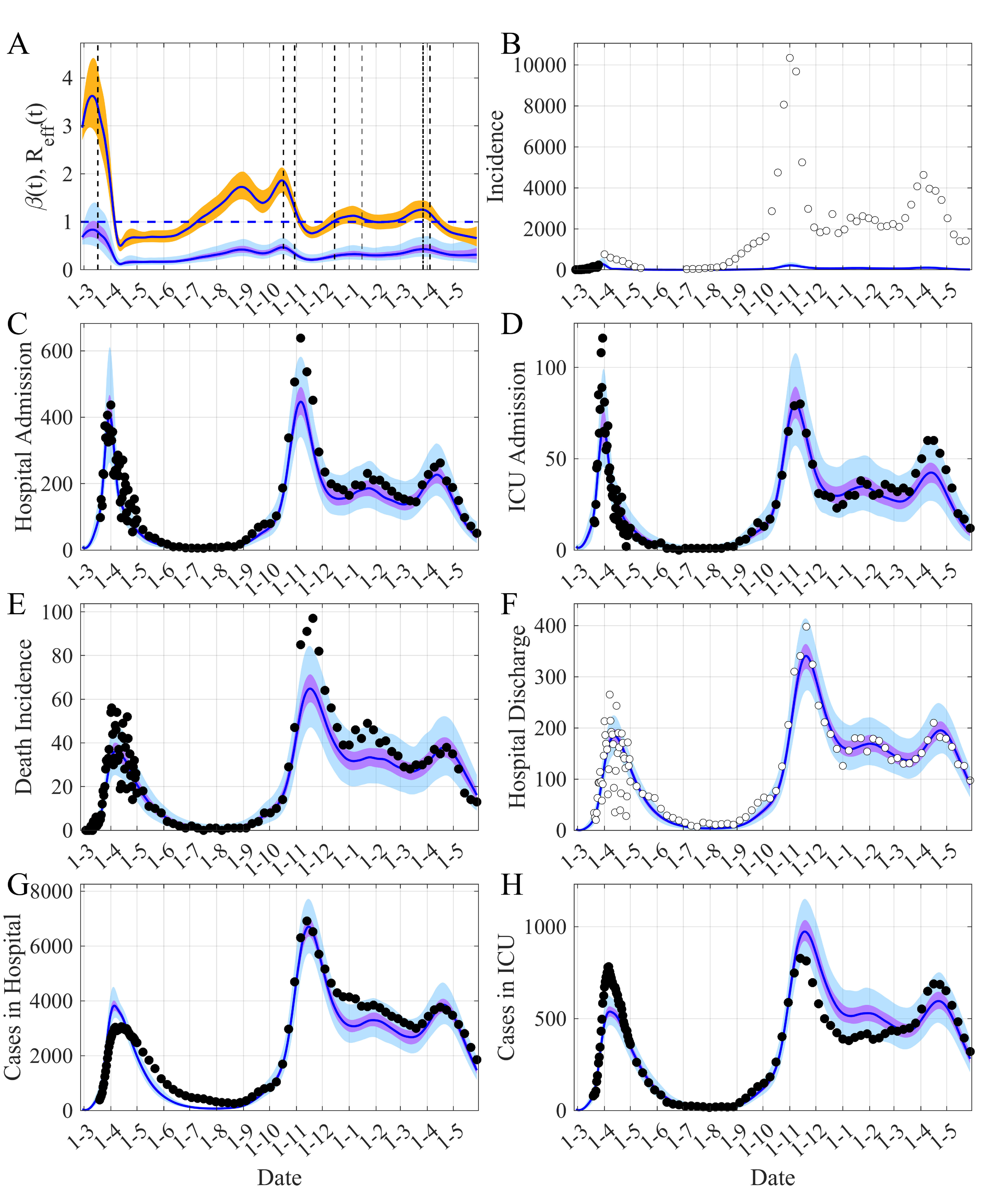

Supplement: S18 Fig — Caption as for Fig 2. The black points are observations used by the inference process, the white points are the observations not used. (TIF) [file pcbi.1009211.s021.tif]

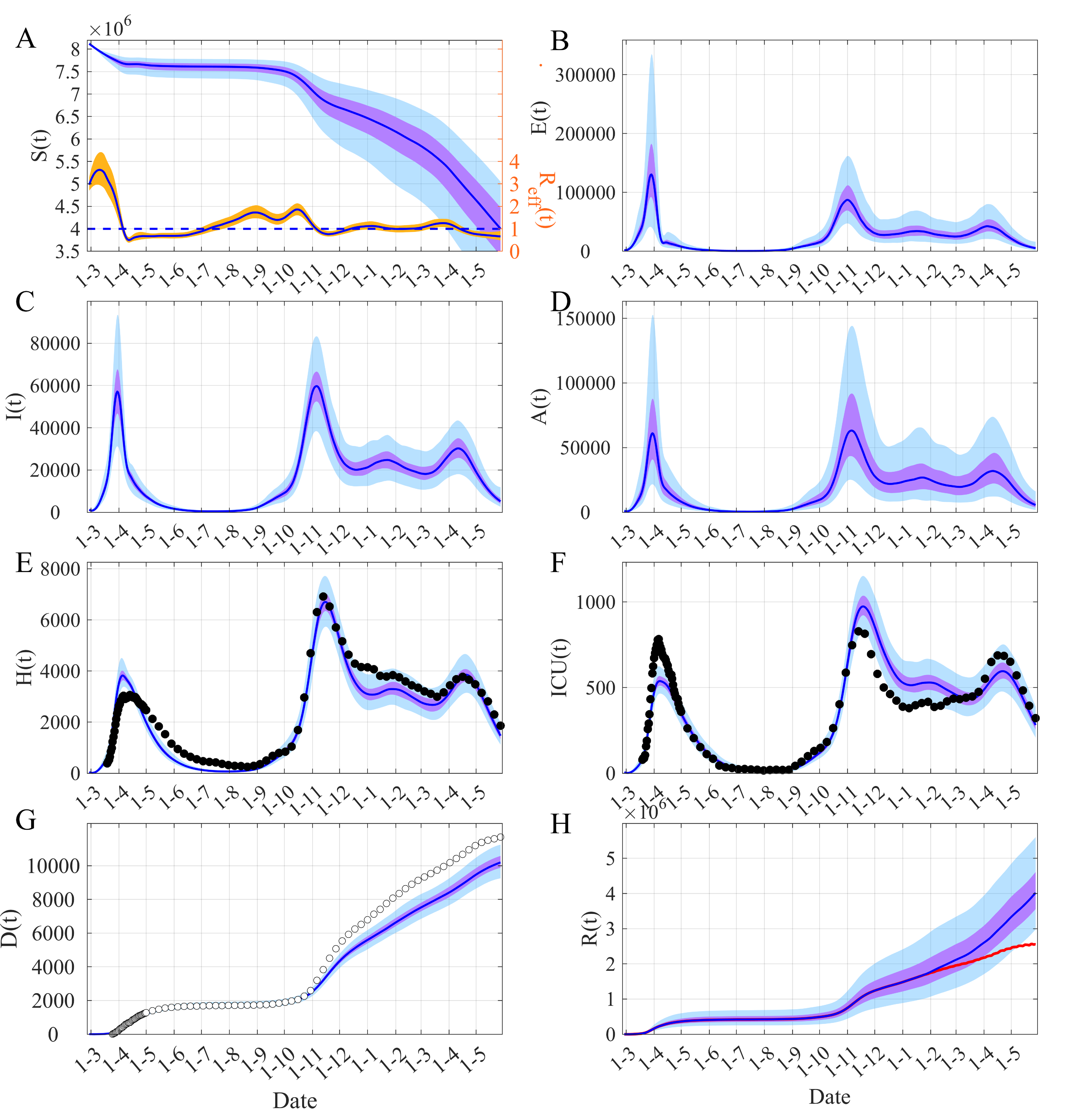

Supplement: S19 Fig — Caption as for Fig 3. The black points are observations used by the inference process, the white points are the observations not used. (TIF) [file pcbi.1009211.s022.tif]

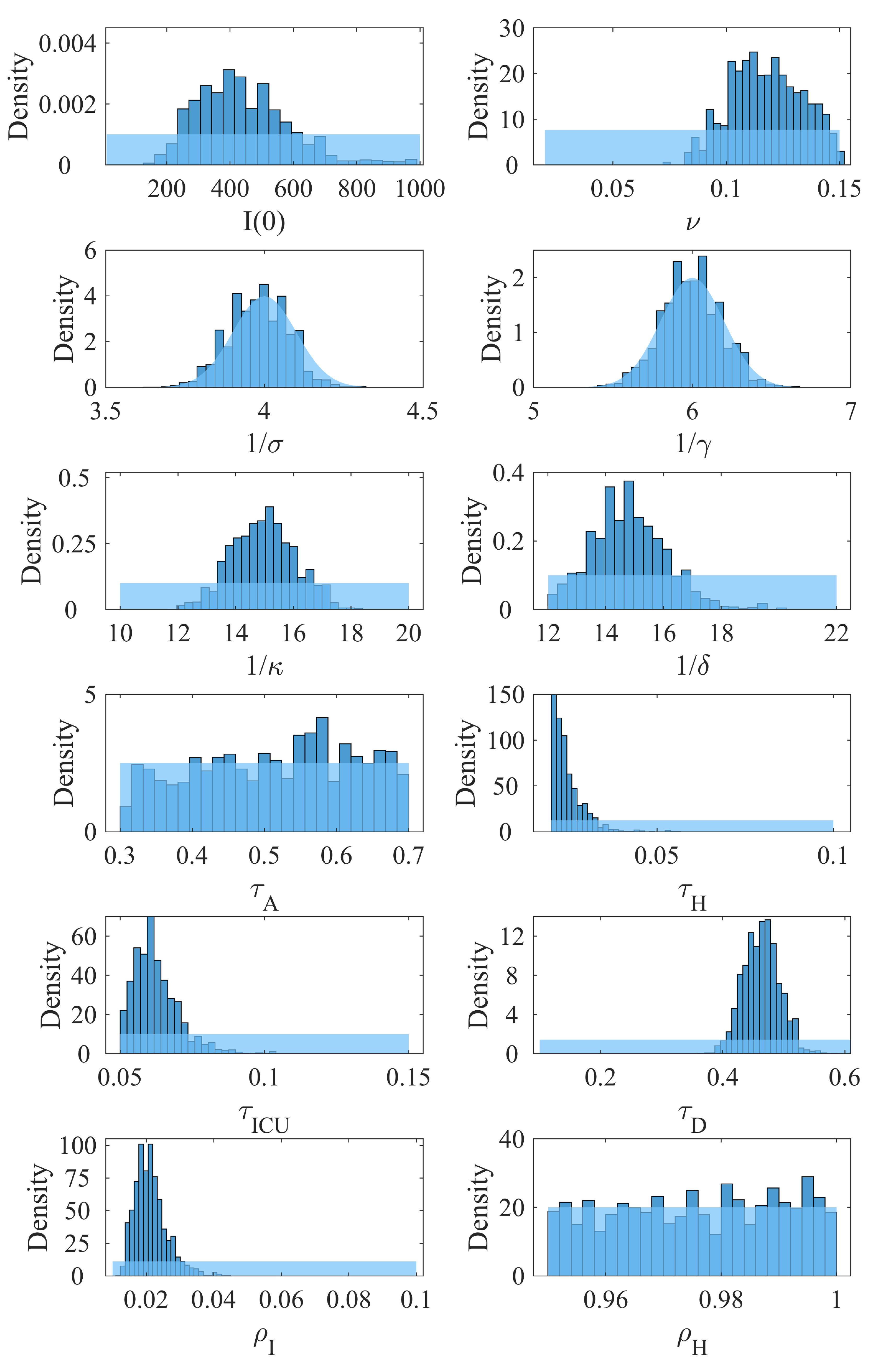

Supplement: S20 Fig — Caption as for S1 Fig. (TIF) [file pcbi.1009211.s023.tif]

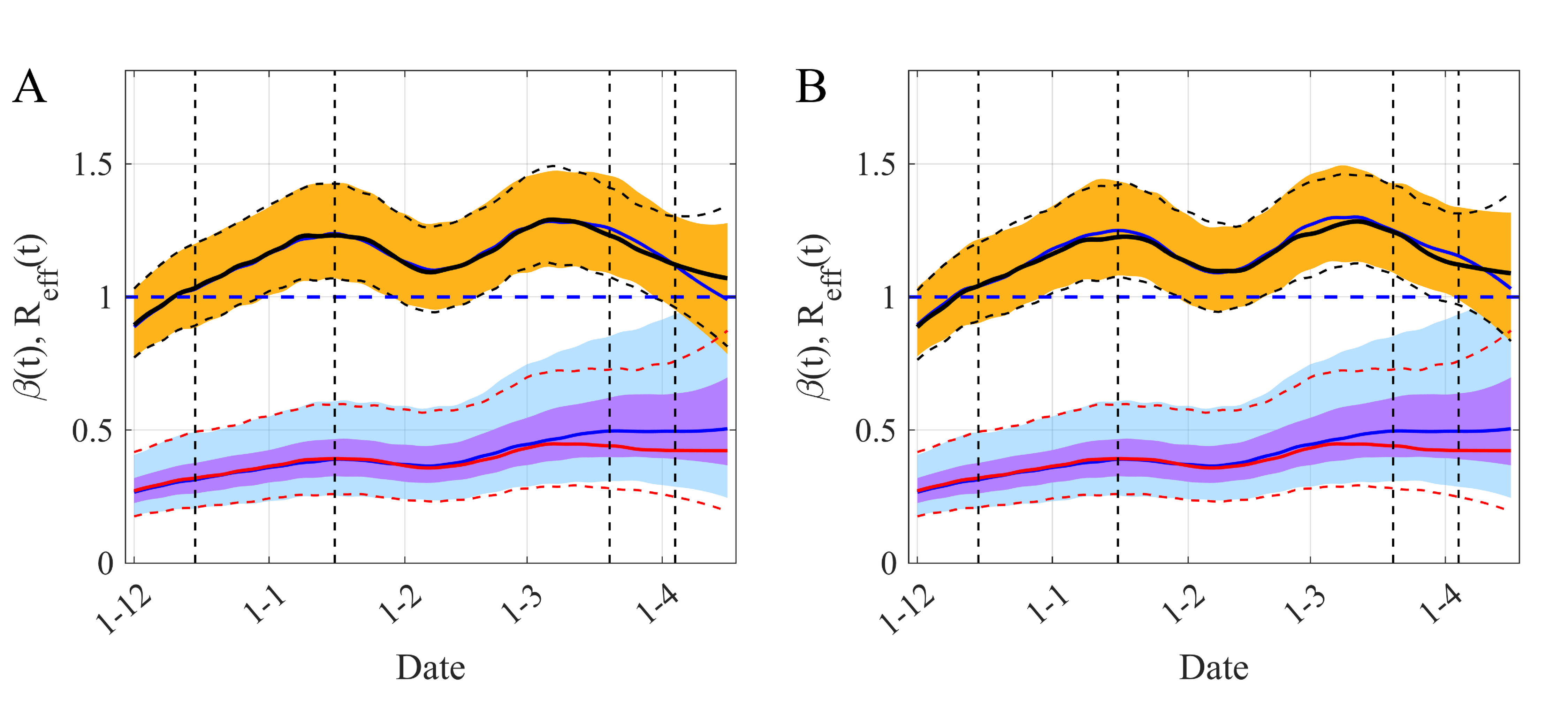

Supplement: S21 Fig — For the case with vaccination, the color areas and blues lines are similar to Fig 2. For the case without vaccination, for β(t) the red line is its median and the dashed red lines are the 95% CI, for Reff(t) the black line is its median and the dashed black lines are the 50% CI. The same observations between 27-02-2020 and 16-04-2021 have been used for these two cases. The vertical dashed lines show the dates of the implementation of the main NPI measures and the horizontal dashed-line the threshold Reff = 1. In (A) the hospital discharges are included in the inference process, whereas in (B) the inference process does not account for them. (TIF) [file pcbi.1009211.s024.tif]

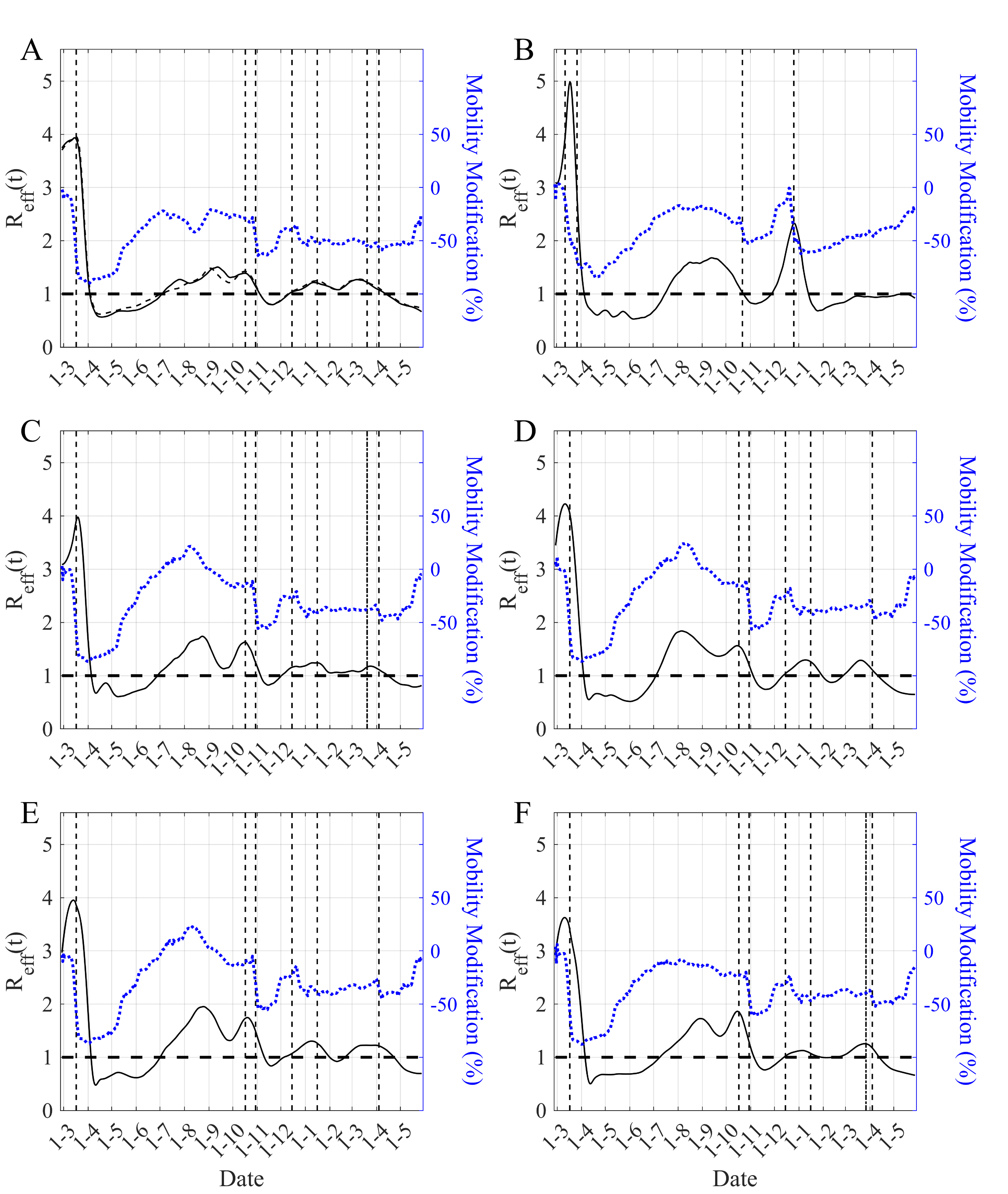

Supplement: S22 Fig — (A) Ile-de-France, (B) Ireland, (C) Provence Alpes Côte d’Azur, (D) Occitanie, (E) Nouvelle-Aquitaine, (F) Auvergne Rhône Alpes. Black line: time evolution of the estimated Reff(t) and blue line: public transport mobility. In (A) the back line corresponds to the case where hospital discharges are included in the inference process, whereas the dashed-line corresponds to the model that does not account for them. The vertical black dashed lines correspond to the start dates of the main mitigation measures, the dot-dashed lines are for cases where only one part of the region has been subjected to these measures. (TIF) [file pcbi.1009211.s025.tif]
