# Supplementary material for: A mechanistic and data-driven reconstruction of the time-varying reproduction number: Application to the COVID-19 epidemic
Source: PLoS Comput Biol. 2021 Jul 26;17(7):e1009211. doi: 10.1371/journal.pcbi.1009211 (PMC8341713; doi:10.1371/journal.pcbi.1009211)
Supplement: Attachment — Submitted filename: PLOS ComputationalBio_article Review Summary.docx [file pcbi.1009211.s026.docx]

**Reviewer’s Comments**

The study focused on the effective reproduction number, R_eff(t)_ , in the context of COVID-19 using a stochastic model of its propagation considering a time-varying transmission rate-a critical parameter. The authors presented well the research gap by summarising the caveats and biases in the R_eff(t)_ not being accounted for in existing literature. The proposed modelling framework is sophisticated as it incorporates many important parameters as well as a time-dependent transmission rate and the stochasticity to imitate the real-world pandemic situation.

The results of the model predictions for selected French and Ireland regions are very enticing and important. They can be clearly related to the impact of lockdown with epidemic and R_eff(t)_ evolution. A big highlight of the results was that the model enables to reconstruct the temporal evolution of transmission rate of COVID-19 using the available data without relying on additional hypothesis of its evolution.

The fact that the study reports new empirical results in Table 1 comes as a surprise. In my view the study would be more attractive if this was clearer from start, ie if already in the abstract the overall % reduction I Reff is given in the first to third wave.

The authors are aware of study limitations and have document them in discussions section. They highlighted the impact of mitigation strategies on COVID-19 dynamics using the modelled R_eff(t)_. The study is timely and could be a very good addition for the policy makers as well as for researchers by enabling the later community to extend it to more regions or countries in future.

A few general comments are given below both for *main text* and for *supporting information*.

**Comments**

**Main Text**

- Equations are difficult to read, some symbols are missing in my version.
- In **Abstract** section, paragraph 2, line 4, abbreviation in bracket (PMCMC) can be removed as it was introduced yet.
- In Results section, paragraph 4: *Fig. 4 also illustrates the robustness and but …*,

this sentence should be written correctly.

- In **Results** section, paragraph 4, line 5-6: dates mentioned should be consistent to the style adopted elsewhere in the text.
- In **Table 1** , 1^st^ column, OC and NA , ‘C’ and ‘O’ are missing, respectively.
- In **Table 1** , 3^rd^ column, date for peak value for each respective region would make more sense and be more informative.
- In section, **‘Modelling a time-varying R_eff_’,** last paragraph: authors should mention explicitly the ‘EpiEstim package’ with a reference as they did for the second method, they mentioned which uses Kalman-filtering.
- In **Discussion** section, paragraph 3, lines 8-10, *‘For the first lockdown we estimated a decrease of around 80% in transmission while between 45 to 55% in French regions and less than 25% in Ireland (Table 1) during the second lockdown, which was less restrictive’.*

The sentence should be re-written correctly make the distinction for first and second lockdown reduction in transmission for France.

- In **Discussion** section, paragraph 6 *: Clearly the weakness of our approach is the dependence of the Reff(t) estimations on…’* the authors begin the paragraph with some sort of limitations of the method, however, between the lines, they highlight and discuss the crucial results of the model.

Similarly, paragraph 7, begins with limitations of the study, (*Our study is not without limitations…).*

It is recommended to reshuffle the text in a way that the limitations of the study are put together in same paragraph whereas the discussion about important results and interpretation could be allocated a separate paragraph.

**Supporting Information**

- In section ***‘Epidemiological Model- hidden state’,*** paragraph2, line 7: *ICI* should be replaced with ICU.
- In section ‘**Observational Model**’ section, the authors assumed a discretized normal distribution for the variables therein. Adding some text on as why the particular distribution was assumed would add more value for the reader to understand. Additionally, consider the same lines of the text :

*We make the assumption that these variables Xk,obs follow a discretized normal distribution with fixed standard deviation proportional to its estimated higher value and with mean equal to the predicted values by the model Xk (equations S2) times an observation rate ..(see Table S1) and with fixed standard deviation proportional to its estimated highest value.*

The above sentence is too long and should be split into few sentences to make it more comprehendible.

- Table S3, column3-5 , parameter $\upsilon$ : U[0.02, 015] probably refers to U[0.02, **0.15**].
- Figures axes labels should be consistent throughout (see **Fig1 A, B** etc. as exceptional cases), use either absolute numbers or exponential notation. In Figure **S10 A**, right y-axis needs to be labeled correctly.
