# Supplementary material for: A mechanistic and data-driven reconstruction of the time-varying reproduction number: Application to the COVID-19 epidemic
Source: PLoS Comput Biol. 2021 Jul 26;17(7):e1009211. doi: 10.1371/journal.pcbi.1009211 (PMC8341713; doi:10.1371/journal.pcbi.1009211)
Supplement: Attachment — Submitted filename: Mechanistic data-driven Reff_Review_Answers.docx [file pcbi.1009211.s027.docx]

Firstly we wish to extend our appreciation to the reviewers for their work and constructive feedback. Please find below the point by point response letter to referees.

**Reviewer #1**

**In this manuscript, authors propose a bayesian framework to reconstruct the temporal evolution of the transmission rate of COVID-19 pandemic using a stochastic model based on Brownian Motion. Authors did a very comprehensive analysis of their results, and I really appreciate the deep level of investigation they carried out. Although I fully support the publication of this manuscript, I believe that it can benefit from certain changes and clarifications to main text.**

**— General comments about the paper**

**- Throughout the text, authors emphasize that their approach has no specific hypothesis on the evolution of the transmission rate. Although they use a diffusion process to model the change in the transmission rate, this does not mean that they do not have any assumptions on it. In fact, they are using a relatively complex SEIR model with many compartments, which intrinsicly imposes assumptions on the dynamics of the disease spread, which are manifested in the parameters defining the flow between these compartments, which are included in Eq. (2).**

We agree with the reviewer. This point is now more clearly mentioned in the revised version of our manuscript.

Now we have amended to:

- in the Abstract: “This mechanistic modeling framework enables us to reconstruct the temporal evolution of the transmission rate of the COVID-19 based only on the available data. Except for the specific model structure, it is non-specifically assumed that the transmission rate follows a basic stochastic process constrained by the observations.”

- in the Method: “Overall, the main advantage of this approach is that it is data-driven. Indeed, in this framework, except for the mechanistic assumptions underlying the model, the estimation of the time-varying parameters, based on the available epidemiological observations [27], is done only under the non-specific assumption that they follow a basic stochastic process.”

- in the Discussion: “This enables us to reconstruct the temporal evolution of the transmission rate of COVID-19 with the non-specific hypothesis that it follows a basic stochastic process constrained by the available data.”

**- Again, regarding the modeling of beta : as far as I see it, there are two things to distinguish here. One is the time-dependent nature of beta, the other is that the derivative of beta being a normal variable. Since the novelty of this manuscript mainly lies in Eq (1), the additional effect of modeling *beta(t)* as Brownian Motion as opposed to a time-independent variable *beta*, or a time-varying *beta(t)* in a different fashion (since there are modeling studies which use beta as a time dependent variable in different ways as well) should be better explained throughout the manuscript.**

In the new version, in several places, we now specify that our beta(t) evolves according to a Browninan motion, following a non-specific hypothesis or using non-specific mechanistic terms, by contrast with models in which deterministic variations are assumed (e.g. beta(t) can be written as a periodic function illustrating weather influence on transmission).

**On the same note, I would suggest that authors re-construct the first paragraph of page 4 (starting on page 3) where they mention the advantages of this approach. Considering the asymptomatic transmission or including the mechanisms or pathogen transmission is not specific to this study, nor the novelty in it. The whole idea of using Brownian motion should be better emphasized to distinguish this work than the others which include similar models and time-dependent variable approaches.**

In this paragraph the aim is to compare our approach for estimating Reff to the currently used methods (such as EpiEstim for example). Then, we think that it’s not exactly true to say that all mechanisms of transmission are incorporated in the classical method for estimating Reff. For instance, with the renewal equation it’s not possible to introduce silent transmission.

This paragraph was modified and we now specify that the comparison is between the methods for estimating Reff: “The advantages of this approach compared to the existing ones consist in (i) the description of the mechanisms underlying pathogen transmission and hence its particularities (asymptomatic phase), (ii) the joint use of multiple datasets (incidence and hospital data), (iii) the explicit taking into account of the uncertainty associated with the data used and especially (iv) the monitoring of the temporal evolution of some of the model parameters without inclusion of external variables.”

**- Brownian process is non-stationary, but the increments of it are stationary (as reflected in the volatility parameter in Eq(1)). Can authors comment on how the value of this parameter effect modeling the sudden changes (such as lockdowns imposed overnight) relative to the time-step of the integration process of the ODE system? In the supplementary information, there are upper and lowerbounds defined for this parameter but the reasoning of these bounds are not really discussed. Are those numbers heuristic? (There is a typo on the upperbound, it should be 0.15, but written as 015)**

This is typically the main topic of our previous paper on this subject that was a sort of proof of concept where we reconstructed known variables of a toy model, for example with a sudden modification of a mixing parameter. Large upper bounds of the volatility allow large fluctuations of beta(t), but these fluctuations are not very smooth. Upper bounds that are small permit large fluctuations of beta(t) but take more time to be effective. So, the upper bound=0.015 is just based on our experience to obtain fluctuations of beta(t) that are not too erratic, within a reasonable range of variation. For instance, the simulation corresponding to the time period November 2020 – April 2021 in IdF does not need large fluctuation of beta(t) to accurately reproduce observations and the peak of the posterior of the volatility is between 0.04 and 0.06 (see “vol” in the figure below).

We have modified the text associated with equation (2) as follows:

“The use of a Brownian process can be viewed as a non-specific hypothesis for the supposed variation of β(t), the volatility ν being a regularized factor. Intuitively, the higher the values of ν the larger the variations in β(t), but smaller ν would induce smoother fluctuations of β(t).”

The typo has been corrected.

**- Why is there no flow between the infectious asymptomatics (A1 and A2) to the infectious symptomatics (I1 and I2) compartments? There is accumulating evidence on pre-symptomatic transmission of COVID-19 as well as asymptomatic - can authors comment on the choice of this way of modeling? On one hand, I realize that this would include even more variables, but the SEIR model is already complex and authors run into identifiabiliy issues already as they mention in the discussion. On the other hand, as authors also mention, there is not enough data to distinguish the asymptomatic versus symptomatic transmission. So I really wonder why authors chose this SEIR structure with this particular complexity.**

We use the classical terminology, E for infected non-infectious, and I for infectious. For most of the diseases individuals in E compartment do not exhibit symptoms. This is not really the case for COVID-19, when transmission can occur even in the absence of symptoms. Thus, we use what is now generally admitted in COVID-19 models, a shorter average duration in the class E. Then, pre-symptomatic transmission is included in the I class (I_1_ for us), see for instance Flaxman et al 2020. This explains why there is no flow between A_i_ and I_i_. Besides, we considered important to explicitly account for asymptomatics (A) class, since these individuals transmit less the disease. So, even if they are generally not concerned by the hospitalization track, they contribute to the dynamics of hospital admissions, that we wanted to describe as accurately as possible, in relation to the data we used for inference. Thus, despite some identifiability issues concerning parameters related to infected and infectious compartments, we considered important to maintain them in the model.

Flaxman S, Mishra S, Gandy A, Unwin HJT, Mellan TA. Coupland H, et al. Estimating the effects of non-pharmaceutical interventions on COVID-19 in Europe. Nature. 2020;584:257-261.

**- There is a confusion about assesing the effect of interventions versus corelating them with the estimated Reff throughout the text. Authors claim that they appropriately asses the effect of mitigation strategies (and then say that they “correlate” certain other NPI with Reff in other parts of the manusctipt). What changes Reff is very multidimensional and depends on many factors, so appropriately assessing any effect by using the changes in Reff in this study is too strong of a claim, since there is no hypothesis on the fact that it will change given the mitigation measures. In that sense, the argument of having a hypothesis-free transmission rate versus using the estimates of it to quantify a certain NPI does sound contradictory (Also mentioned in the discussion, which should be fixed).**

For us, like many others, Reff provides an estimation of the transmission in the population and at least can be used as a proxy for the transmission potential of a pathogen. Then, we can use it to try to explain the observed course of an epidemic.

For the specific case of the COVID-19 in the settings studied we just highlight that the concomitance between the abrupt reduction in the transmission and the implementation of the mitigation measures may suggest a potential link. This is also true that we did not explore other hypothesis for the end of the first wave while the susceptibles are not depleted.

Whether it's directly part of the model, or whether you're looking at an a posteriori correlation, either way it is only correlative and not causal, but it is difficult do better in a population-based study.

We have been careful not to go too far in concluding this link between epidemic decrease and effect of NPI.

Page 6: “In both cases, given the temporality of the decline compared to the timing of the NPIs, these sharp decreases seem to be the result of the implementation of the mitigation measures.”

Page 8: “Its time evolution allows us to follow, almost in real time, the evolution of the course of the epidemic, and to get information regarding the potential effects of mitigation measures taken”

Page 9: “These reductions in transmission may reflect the nature of the mitigation measures implemented in both countries”

Page 9: “We also found other interesting results such as a significant high correlation between the trend of mobility and our estimation of the transmission between the epidemic waves (see S22 Fig. and [34]),” (This has already been reported in several papers in the top journals.)

Now we rewrote the sentence in the Abstract as: “This approach allows us to follow both the course of the COVID-19 epidemic and the temporal evolution of its R_eff_(t). Besides, it allows to assess and to interpret the evolution of transmission with respect to the mitigation strategies implemented to control the epidemic waves in France and Ireland.”

**- Why data from France and Ireland are used only? I am not asking for more analysis for different countries here, since there is no end to that. But would be useful to know why.**

We have modified the end of the last paragraph in the Introduction as:

“To compare different geographical settings with similar population size, we have chosen to illustrate our approach with data from several regions of France and Ireland.”

**- Authors use the “silent” transmission and “asymptomatic” transmission in many parts of the manuscript. It would be good to define what “silent” transmission means exactly. Do they also include the symptomatic transmissions that are not reported / or asymptomatic transmissions that are reported? What does that really include?**

As done in many COVID-19 models, we have reduced the duration in the class E (infected but not infectious) and increased the duration in the class I (infectious). In this way, we can consider the transmission by infected persons but who did not develop symptoms (see for instance Di Domenico et al 2020).

In the “Supporting Information” we have added the following sentence to the model description:

“As in previous models (*e.g.* Di Domenico et al, 2020; Fergusson et al, 2020), we reduce the incubation period duration to account for pre-symptomatic infectious. In this way individual that become infectious before the end of the infectious period but remain either asymptomatic or develop symptoms are put in the infectious compartment.”

In the main text, when we talk about “silent transmission” (a term shorter than “asymptomatic and pre-symptomatic transmission”), it is to generalize what is discussed in the literature.

- Di Domenico L, Pullano G, Sabbatini CE, Boëlle PY, Colizza V. Impact of lockdown on COVID-19 epidemic in Ile-de-France and possible exit strategies. BMC Medicine. 2020;18:240.

**- Regarding the uncertainities in the generation time and the serial interval (Page 3) - do the authors refer to aleatoric (as those variables being random variables with a certain distribution) or epistemic (as the measurement errors and the variability across different studies) uncertainity here?  This should be better explained troughout the text where the word “uncertainity” is used for other variables as well.**

When we used the term “uncertainty” is mainly to characterize errors and variability associated with a given observed quantity (so measurement errors and the variability across different studies).

In the paragraph that you quote, now we use “variability” to stress that there are numerous studies that have estimated these quantities for the SARS-Cov-2 and there are numerous different results:

“The uncertainty is not exclusively related to the incidence data, there is also a large variability in the value proposed in the literature for generation time or serial interval distributions and average values [15-16].”

We also tried to be more consistent across throughout the text.

**- Authors compare their results with 1) results from EpiEstim package, and 2) the study by Arroyo-Marioli et al. Although I appreciate that the results are compared, these two studies use models that have different complexity (one is based on purely the incidence data, and the other is a simpler SIR model compared to the authors’ comples SEIR model). This should be mentioned in the text, since the differences in estimation accuracy cannot be attributed to the Bayesian approach or the Stochastic beta used in this study solely, but is a combination of both the differences in model complexity, estimation methods, and the incorporated data (In the discussion -> “These differences may be explained by the fact that the two other methods computed their estimations based on the new cases only” - this should be better discussed). On the same note, there are a lot of other studies on estimating Reff - a numerical comparison is not necessary for each, but more of these studies can be included in the main text to give a broader view on the state of the current research to the reader.**

We agree, with this point. We have modified the paragraph in the Discussion as suggested:

“We have also compared the trend of our estimations of *R_eff_* with those of two other methods from the literature [14,30] on data from Ireland (Fig. 5). One of the differences between our estimations and the two others is a greater variability of our *R_eff_* estimations due to an underlying complex mechanistic model used and to uncertainties in the transmission rate, asymptomatic transmission and in the different delays needed for describing hospital multiple datasets. A second difference consists in asynchronous peaks of *R_eff_*. For instance, the peak of *R_eff_* occurs at the beginning of August for the two other methods, while our estimates suggest a peak in mid-September (Fig. 5). These differences may be explained by the differences in model complexity but, above all, by the fact that the two other methods computed their estimations based on the new cases only, which are data subject to certain bias [6-8].”

**- “Taking account of the large variability in the daily observations, since the 1st of May for French data and the 1st of June for Irish data, we have used a weekly average of the observed daily values.” - Why? Isn’t the idea of using Brownian motion for beta is exactly to deal with this variability? Can authors comment on that?**

The main idea also was to average the weekly component (likely due to the week-end component).

With the Brownian process, beta(t) can change each day day to adapt to each new data point, but more smooth estimations seem to us preferable. Then averaging data over the week allows this double objective, obtaining smooth estimation and removing the weekly component.

We re-wrote the sentence in the Data paragraph as:

“We also used cases both in hospital and in ICU. Taking account for the large variability in the daily observations and in particular for an important weekly component, since the 1st of May for French data and the 1st of June for Irish data, we have used a weekly average of the observed daily values.”

**- Discussion (Page 9, Paragraph 1) : “[…] These differences may be explained by the fact that the two other methods computed their estimations based on the new cases only. “ - Given the differences in the number of compartments used in these methods, delays between the peaks in different signals will be different - so can’t this difference in asynchronous peaks regarding other studies be attributed to the general structure of the transmission models they use as well?**

For us the main explanation for this difference is linked to the representativeness of the incidence data in this special epidemic. This representativeness can be estimated by the case reporting rate or case detection rate.

In France (as in many countries we suspect), the reporting and detection rates have fluctuated from very low values to more reasonable values. Pullano et al have estimated that the detection rate increased from 7% in mid-May to 40% by the end of June, compared to well below 5% at the beginning of the epidemic. A more recent study by Hozé et al estimated a detection rate based on surveillance of 40.2% in June to August 2020, 62.3%, in September to October, and 49.3% in November 2020 to January 2021.

As incidence data better describes the reality of the infection in the population (after November 2020) the difference between the different methods is fading away (see the new Fig. 5).

Pullano G, Di Domenico L, Sabbatini CE, Valdano E, Turbelin C, Debin M, Guerrisi C, Kengne-Kuetche C, Souty C, Hanslik T, Blanchon T, Boëlle PY, Figoni J, Vaux S, Campèse C, Bernard-Stoecklin S, Colizza V. Underdetection of cases of COVID-19 in France threatens epidemic control. Nature. 2021;590:134-139.

Hozé N, Paireau J, Lapidus N, Tran Kiem C, Salje H, Severi G, Touvier M, Zins M, de Lamballerie X, Lévy-Bruhl D, Carrat F, Cauchemez S. Monitoring the proportion of the population infected by SARS-CoV-2 using age-stratified hospitalisation and serological data: a modelling study. Lancet Public Health. 2021:S2468-2667(21)00064-5.

**- Tables S1-S3 : Can authors provide the basis for the upper / lower bounds of the prior distributions they use in their study? Are they adopted from other studies or heuristically defined?**

Done

**- Fig S2 : It is a better practice to show multiple chains to demonstrate that the chains are mixing properly.**

We agree, but considering our computational power and the CPU time needs for estimating the parameters for more than 410 days for 6 settings or models, it’s difficult to run two or more chains. Nevertheless it is quite classical to test the convergence on just one chain as proposed by the Geweke diagnosis in the coda package using the first and the last part of the chain.

Geweke J. Evaluating the accuracy of sampling-based approaches to calculating posterior moments. In Bernado JM, Berger JO, Dawid AP, Smith AFM, editors. Bayesian Statistics 4. Clarendon Press, Oxford, UK; 1992.

**- Figs S6-S9-S12-S15-S18 : Due to the dynamics of the SEIR model, there is naturally a second peak in the incidence data estimated by the model correlating with the second peaks in other signals (death, hospital, ICU). This second peak is not observed in the data, and it seems like a re-occuring issue for all the regions / countries authors included in their analysis - nevertheless, this is not discussed in the discussion section (only briefly for Fig S6). Can this be attributed to differences in testing only for 5 different regions simultaneously.**

In the previous dataset used for France, there were just 3 peaks in the hospital data at the beginning of April, in November-December and in January. In the incidence data there were also 3 peaks, in April, in November-December and in January. For Ireland it is more or less similar except that the first hospital peak was at the beginning of April while the first incidence peak was mid-April at least 2 weeks after the hospital peak. So it’s difficult to understand what is the second peak you mention.

As we stressed in the data section we did not use incidence data except for the first part of March 2020 when hospital related data were not available. Thus the dynamics of our model is mainly driven by the hospital data. We think that there is serious bias in the incidence data at the beginning of the epidemic. This bias could be induced by the non-availability of the tests but also by the behavior of people that changed according to their willingness to be tested based on the information received and the obligations imposed by the governments.

Thus the first peaks that we observe in all the studies in April 2020 do represent the real amount of the infections and we did not take it into account. This bias is even more accentuated in Ireland with a delay of more than 15 days between the peak in hospital and the peak of incidence. The incidence data after November appears more coherent with hospital data in the two countries.

In France, the fluctuation of the reporting or detection rate can be illustrated in a paper by Pullano et al that we quoted. These authors have estimated that the detection rate increased from 7% in mid-May to 40% by the end of June, compared to well below 5% at the beginning of the epidemic. With our framework we estimate, for the beginning of March that the detection rate was around 2.5%-5% depending on the regions. A more recent study by Hozé et al estimated a detection rate by surveillance of 40.2% in June to August 2020, 62.3%, in September to October, and 49.3% in November, 2020, to January 2021.

**- Although not necessary, I would strongly recommend the authors to provide a public repository of their work for reproducibility purposes.**

Done**:** https://www.dropbox.com/s/ja84ewdp59jn45k/ssm_seiar_idf.zip?dl=0

**— Wording**

**- PMCMC is an abbreviation and the full name should be used first before the abbreviation itself.**

Done

**- […] which measures how many amore people -> how many *more* people (I would recommend to use line numbers next time, makes it easier for both the reviewers and the authors.)**

Done

**- “multiple hospital datasets” may sound better than “hospital multiple datasets”.**

Done

**Review #2**

**The study focused on the effective reproduction number, R_eff(t)_ , in the context of COVID-19 using a stochastic model of its propagation considering a time-varying transmission rate-a critical parameter. The authors presented well the research gap by summarising the caveats and biases in the R_eff(t)_ not being accounted for in existing literature. The proposed modelling framework is sophisticated as it incorporates many important parameters as well as a time-dependent transmission rate and the stochasticity to imitate the real-world pandemic situation.**

**The results of the model predictions for selected French and Ireland regions are very enticing and important. They can be clearly related to the impact of lockdown with epidemic and R_eff(t)_ evolution. A big highlight of the results was that the model enables to reconstruct the temporal evolution of transmission rate of COVID-19 using the available data without relying on additional hypothesis of its evolution.**

**The fact that the study reports new empirical results in Table 1 comes as a surprise. In my view the study would be more attractive if this was clearer from start, ie if already in the abstract the overall % reduction I Reff is given in the first to third wave.**

As our initial focus was on the methodological aspects, we have chosen not to highlight too much the empirical results. In the revised version we try to be more explicit since the beginning in the Abstract: “This approach allows us to follow both the course of the COVID-19 epidemic and the temporal evolution of its *R_eff_(t)*. Besides, it allows to assess and to interpret the evolution of transmission with respect to the mitigation strategies implemented to control the epidemic waves in France and in Ireland. We thus can estimate a reduction of more than 80% for the first wave in all the studied regions but a smaller reduction for the second wave when the epidemic was less active. For the third wave in Ireland the reduction was again significant (>70%).”

**The authors are aware of study limitations and have document them in discussions section. They highlighted the impact of mitigation strategies on COVID-19 dynamics using the modelled R_eff(t)_. The study is timely and could be a very good addition for the policy makers as well as for researchers by enabling the later community to extend it to more regions or countries in future.**

**A few general comments are given below both for *main text* and for *supporting information*.**

**Comments**

**Main Text**

- **Equations are difficult to read, some symbols are missing in my version.**

We have double-checked all the symbols in the main text.

- **In Abstract section, paragraph 2, line 4, abbreviation in bracket (PMCMC) can be removed as it was introduced yet.**

Done

- **In Results section, paragraph 4: *Fig. 4 also illustrates the robustness and but …*,**

**this sentence should be written correctly.**

Done

- **In Results section, paragraph 4, line 5-6: dates mentioned should be consistent to the style adopted elsewhere in the text.**

Corrected

- **In Table 1, 1^st^ column, OC and NA , ‘C’ and ‘O’ are missing, respectively.**

This problem could be due to printing issue, there is no any character missing in our electronic version.

- **In Table 1 , 3^rd^ column, date for peak value for each respective region would make more sense and be more informative.**

The dates of the first peak of R_eff_ for each region have been added.

- **In section, ‘Modelling a time-varying R_eff_’, last paragraph: authors should mention explicitly the ‘EpiEstim package’ with a reference as they did for the second method, they mentioned which uses Kalman-filtering.**

Done

- **In Discussion section, paragraph 3, lines 8-10, *‘For the first lockdown we estimated a decrease of around 80% in transmission while between 45 to 55% in French regions and less than 25% in Ireland (Table 1) during the second lockdown, which was less restrictive’.***

**The sentence should be re-written correctly make the distinction for first and second lockdown reduction in transmission for France.**

Done, we have re-written these sentences as:

“For the first lockdown, we estimated a decrease of around 80% in transmission in both countries. For the second wave, our reduction estimations were between 45 to 55% in French regions and around 20% in Ireland (Table 1). While France is undergoing another major epidemic wave, we have estimated a reduction of more than 70% of the transmissibility of the third wave in Ireland (Table 1). These reductions in transmission may reflect the nature of the mitigation measures implemented in both countries. For the second wave, these measures were less restrictive than during the first wave, nevertheless the second wave was also less severe. In Ireland, the mitigation measures introduced in the third wave were similar to the first wave.”

- **In Discussion section, paragraph 6 *: Clearly the weakness of our approach is the dependence of the Reff(t) estimations on…’* the authors begin the paragraph with some sort of limitations of the method, however, between the lines, they highlight and discuss the crucial results of the model.**

**Similarly, paragraph 7, begins with limitations of the study, (*Our study is not without limitations…).***

**It is recommended to reshuffle the text in a way that the limitations of the study are put together in same paragraph whereas the discussion about important results and interpretation could be allocated a separate paragraph.**

This paragraph was not intended to be a paragraph where we cite some limitations of our approach but, as you stressed, we wanted to highlight our model performance despite its simplicity. We agree that the beginning of the first sentence were awkward. We rewrote that sentence as:

“The main characteristic of our approach is the dependence of the *R_eff_(t)* estimations on the relevance of the underlying mechanistic model and the accuracy and completeness of the available data.”

**Supporting Information**

- **In section *‘Epidemiological Model- hidden state’,* paragraph2, line 7: *ICI* should be replaced with ICU.**

Done

- **In section ‘Observational Model’ section, the authors assumed a discretized normal distribution for the variables therein. Adding some text on as why the particular distribution was assumed would add more value for the reader to understand. Additionally, consider the same lines of the text:**

We make the assumption that these variables Xk,obs follow a discretized normal distribution with fixed standard deviation proportional to its estimated higher value and with mean equal to the predicted values by the model Xk (equations S2) times an observation rate (see Table S1) and with fixed standard deviation proportional to its estimated highest value.

**The above sentence is too long and should be split into few sentences to make it more comprehendible.**

We rewrote this sentence and added a reference for the discretized normal distribution in a similar context, an assumption quite often used for epidemic related data different from the incidence of the number of cases.

Davies NG, Barnard RC, Jarvis CI, Russell TW, Semple MG, Jit M, Edmunds WJ; Centre for Mathematical Modelling of Infectious Diseases COVID-19 Working Group; ISARIC4C investigators. Association of tiered restrictions and a second lockdown with COVID-19 deaths and hospital admissions in England: a modelling study. Lancet Infect Dis. 2021;21:482-492. doi: 10.1016/S1473-3099(20)30984-1.

- **Table S3, column3-5, parameter** $\boldsymbol{\upsilon}$ **: U[0.02, 015] probably refers to U[0.02, 0.15].**

Done

- **Figures axes labels should be consistent throughout (see Fig1 A, B etc. as exceptional cases), use either absolute numbers or exponential notation. In Figure S10 A, right y-axis needs to be labeled correctly.**

Unfortunately it's a constraint imposed by Matlab that is used for the figures and we didn't manage to bypass it. Fig. S10A right y-axis has been corrected.
