# Supplementary material for: A mechanistic and data-driven reconstruction of the time-varying reproduction number: Application to the COVID-19 epidemic
Source: PLoS Comput Biol. 2021 Jul 26;17(7):e1009211. doi: 10.1371/journal.pcbi.1009211 (PMC8341713; doi:10.1371/journal.pcbi.1009211)
Supplement: Attachment — Submitted filename: Mechanistic data-driven Reff_Review_Answers_V3.docx [file pcbi.1009211.s028.docx]

Firstly we wish to extend our appreciation to the reviewers for their work and constructive feedback. Please find below the point by point response letter to reviewers.

Reviewer #1: I would like to thank the authors very much for addressing all my questions. The main text is properly adjusted, and in my opinion reads much better now.

I only have one more suggestion - not mandatory but I think it would be better for the sake of scientific rigor. Since we had the discussion on how many markov chains are used for convergence diagnostics, it can be very useful if authors provide the configurations of their simulations in the supplementary material - such as how many chains are used per realization, what is the burn-in period, what is the number of jumps per chain, what is the dilution, and as the authors mention themselves, what are the diagnostics (such as geweke criterion etc.)?

*Done*

*In the Supplementary Material:*

*Then, the adaptive PMCMC is run on the output of the KMCMC. For each region or country just one chain of PMCMC has been run with p particles m iterations for the chain. Depending on the convergence velocity, we have used p=2000 or p=3000 particles with m=100,000 or m=200,000 iterations. Each time the burning is equal to 20% of the length of the analyzed chain m. In order to assess convergence of the chains, the visual inspection of the chains (see S2 Fig.) was complemented by Geweke diagnosis (Geweke, 1992), the parameter means computed using the first 20% and the last 50% of the chain are compared through a Z-score.*

*In the S2 Figure caption:*

*The acceptance rate is equal to 13.6% and the chain is stationary, ie stationarity is not rejected at the 5% level by the Geweke diagnosis*

*Geweke, J. Evaluating the accuracy of sampling-based approaches to calculating posterior moments. In Bernado JM, Berger JO, Dawid AP, Smith AFM, editors. Bayesian Statistics 4. Clarendon Press, Oxford, UK;1992.*

Reviewer #2: Except for a minor comment in the abstract section, as a reader, the current version of the manuscript is according to my expectations, and I recommend it for publication.

In the abstract, please consider making modifications to the following lines:

“We thus can estimate a reduction of more than 80% for the first wave in all the studied regions but a smaller reduction for the second wave when the epidemic was less active. For the third wave in Ireland the reduction was again significant (>70%).”

It would be consistent in writing style as well as more informative for the reader who just reads the abstract only to represent the reduction amount for second wave using a percentage of point estimate just like for the first and third waves.

*Done*

*“We can thus estimate a reduction of more than 80% for the first wave in all the studied regions but a smaller reduction for the second wave when the epidemic was less active, around 45% in France but just 20% in Ireland. For the third wave in Ireland the reduction was again significant (>70%).”*

Have the authors made all data and (if applicable) computational code underlying the findings in their manuscript fully available?

*Yes, see:* ***S1 Code and data used:*** *https://www.dropbox.com/s/ja84ewdp59jn45k/ssm_seiar_idf.zip?dl=0*
